# Transcriptome Analysis of Hepatitis C Virus-Infected Hepatoma Cells Following Stringent Removal of Genomic DNA

**DOI:** 10.3390/v18101167

**Published:** 2026-10-20

**Authors:** Mozhdeh Khajouei, Elena Jost, Maximilian Collatz, Attiya Qadoos Malik, Gesche K. Gerresheim, Nina Hofmann, Pan Hu, Nikolai Grahn, Ragna Weitz, Jan Dreute, Lena Soppa, Alexander Goesmann, Dieter Glebe, Manja Marz, Michael Niepmann

**Affiliations:** 1 Institute of Biochemistry, Faculty of Medicine, Justus-Liebig-University, 35392 Giessen, Germany; khajoueimozhdeh@gmail.com (M.K.); jost.elena@web.de (E.J.); qadoos@gmx.de (A.Q.M.); gesche.gerresheim@staff.uni-marburg.de (G.K.G.); n.grahn@web.de (N.G.); ragna.weitz@web.de (R.W.); jan.dreute@biochemie.med.uni-giessen.de (J.D.); 2 RNA Bioinformatics and High-Throughput Analysis, Friedrich Schiller University, 07743 Jena, Germany; maximilian.collatz@uni-jena.de (M.C.); manja@uni-jena.de (M.M.); 3 Bioinformatics Core Facility Jena, Friedrich Schiller University, 07743 Jena, Germany; 4 Bioinformatics and Systems Biology, Justus-Liebig-University, 35392 Giessen, Germany; nina.hofmann@computational.bio.uni-giessen.de (N.H.); alexander.goesmann@computational.bio.uni-giessen.de (A.G.); 5 National Emerging Infectious Diseases Laboratories (NEIDL), Department of Virology, Immunology, and Microbiology, Boston University Medical School, Boston, MA 02118, USA; cannyhp@126.com; 6 Institute of Medical Virology, Justus-Liebig-University, National Reference Center for Hepatitis B and D Viruses, Giessen, and German Center for Infection Research (DZIF), Partner Site Giessen, 35392 Giessen, Germanydieter.glebe@viro.med.uni-giessen.de (D.G.)

**Keywords:** *Lnc-ITM2C-1*, *GCSIR*, lncRNA, *GPR55*, TRIzol, gDNA, RNA-seq, NGS

## Abstract

Hepatitis C Virus (HCV) is a positive strand RNA virus that infects hepatocytes and reprograms cellular gene expression, contributing to chronic liver disease and hepatocellular carcinoma. HCV replicates slowly and induces limited changes in the cellular transcriptome. Here, we improved analysis of low-abundance transcripts, including long non-coding RNAs (lncRNAs), in the context of HCV replication. We stringently removed genomic DNA from total RNA from Huh-7 hepatoma cell lines by a combination of TRIzol, acidic phenol, silica matrix columns and recombinant DNase I. RNA sequencing (RNA-Seq) and targeted validation were used to characterize *Lnc-ITM2C-1* splice variants and expression of its neighboring gene, G protein-coupled receptor 55 (*GPR55*), to investigate their potential roles in HCV replication. Challenging our previous findings, under our improved experimental conditions, *Lnc-ITM2C-1* and *GPR55* were neither involved in a positive regulation circuit with HCV nor induced detectable transcriptome changes. Secondary analysis of RNA-Seq data according to HCV infection status revealed statistically robust HCV-induced expression of 21 host cell genes involved in pathways like stress response (in particular ATF3), lipid metabolism, cytoskeleton organization and growth regulation. In contrast, we found that expression changed for only a limited subset of previously reported HCV-associated lncRNAs (e.g., EGOT). This highlights the necessity of stringent gDNA removal for analysis of low-abundance transcripts like lncRNAs.

## 1. Introduction

Hepatitis C virus is a plus-strand RNA virus that infects the liver [1,2,3]. After infection of a cell, the virus establishes replication complexes in membrane organelles derived from the endoplasmic reticulum (ER), the so-called membranous web, in order to replicate its plus strand RNA genome via a minus strand intermediate [4,5,6,7]. Fully infectious HCV genomes can go through all steps of a complete infection cycle in cell culture, using either natural HCV isolates [8,9,10] or a chimera like the efficiently replicating Jc1 clone [11].

In contrast to many other viruses that replicate within a few hours in their host cells, HCV replicates rather slowly. The maximum of viral RNA replication is reached only after about two days [12,13,14,15]. Many other viruses reach this maximum much earlier, some of them already after 5 h [14,15]. Concurrently, the amount of HCV RNA molecules in an infected cell is also lower compared with other viruses. HCV replication yields not more than about 10^4^ RNA molecules of plus strand RNA per cell in cell culture [12,13,15] and even less in hepatocytes of infected patients [16], whereas Picornaviruses like Coxsackievirus B3 or Poliovirus produce about 10^6^ molecules per cell [14,15].

Although HCV can cause fulminant acute infection [8], infections often remain inapparent and undiagnosed. About 70% of HCV infections become chronic, often resulting in liver cirrhosis and liver cancer [17,18,19]. Establishment of such chronic infection is usually accompanied by viral evasion from innate and adaptive immune responses [18,20,21], likely supported by the production of quasispecies progeny genomes by the error-prone viral replicase [22,23]. Moreover, HCV infection causes changes in cellular gene expression towards metabolic reprogramming [24,25,26,27,28,29,30] and the development of cancer [17,18,19].

Despite the above important changes in cellular gene expression after HCV infection, the overall extent of gene expression changes in HCV-infected cells appears to be limited to only a few dozen [27] or a few hundred detected differentially expressed genes (DEGs) [31,32,33,34,35], with gene numbers depending on the time after infection, the experimental settings and on the stringency of fold-change and significance thresholds. In contrast, some negative-strand viruses change the expression of about 2000 to 6000 genes within 12 to 24 h after infection [36]. Apparently, the lower number of cellular DEGs in HCV-infected cells is consistent with the slower and lower-level replication of the virus.

In the context of HCV replication, we had previously reported that a long non-coding RNA (lncRNA), namely *Lnc-ITM2C-1*, and its neighbor gene encoding G protein-coupled receptor 55 (*GPR55*), are differentially expressed in HCV-infected cells [37]. Moreover, we had hypothesized that *Lnc-ITM2C-1* and *GPR55* mRNA are involved in a positive regulation circuit during HCV infection, involving the stimulation of *Lnc-ITM2C-1* expression by HCV, followed by the stimulation of *GPR55* mRNA expression by *Lnc-ITM2C-1*. *GPR55* then suppresses Interferon Stimulated Gene (ISG) expression, thereby increasing permissiveness to HCV replication.

To further investigate our previously proposed *Lnc-ITM2C-1*—*GPR55* regulatory circuit in more detail, we aimed to identify which of the splice variants of *Lnc-ITM2C-1* RNA and *GPR55* mRNA are expressed in Huh-7 hepatoma cells and therefore are the molecules in action. However, in our initial attempts to detect such *Lnc-ITM2C-1* and *GPR55* splice variants and their differential regulation upon HCV infection, we encountered difficulties to reproduce our former results [37]. Also, other studies had reported strong detrimental effects on the reliable analysis of the expression of lncRNAs, caused by the contamination of total RNA by residual genomic DNA (gDNA) [38,39,40]. Low amounts of gDNA may contaminate different samples in an experiment to extents varying from sample to sample, depending on possible carry-over of contaminating gDNA during total RNA preparation, and may lead to false-positive results in the qPCR reaction that is performed after the reverse transcription step. In our initial experiments, amplification in the “noRT” control (a control reaction to which no Reverse Transcriptase enzyme had been added) for some primer sets raised concerns that residual gDNA contamination could contribute to the measured qPCR signal, especially for low-abundance genes. Such disturbances by false-positive signals caused by residual gDNA are relatively more relevant for low-abundance transcripts. This observation motivated a systematic reassessment of the previously proposed regulatory axis, together with a refined protocol for the extraction of highly purified total RNA from which gDNA has been stringently removed.

The results of the present study were, on the one hand, surprising to us since under the largely improved RNA purification and gDNA removal conditions, we did not confirm the positive stimulation circuit involving HCV, *Lnc-ITM2C-1*, *GPR55* mRNA and ISGs. On the other hand, we reliably found differential expression of several genes involved in pathways relevant to HCV replication, including several low abundantly expressed genes. Moreover, using the above improved RNA purification, we also confirmed differential expression of some other lncRNAs that had been reported before to be differentially regulated during HCV replication, whereas others could not be confirmed in our experimental settings. Thus, our improved RNA purification and gDNA removal protocol may have important implications for future research on low-abundance expressed genes, in particular lncRNAs.

## 2. Materials and Methods

### 2.1. Plasmids and In Vitro Transcription

The HCV full-length RNA genome (J6/JFH1 chimeric genotype 2a) was in vitro*-*transcribed from plasmid pFK-JFH1-J6 C-846_dg (in short: “Jc1”) [11] with T7 RNA polymerase as described previously [37]. The HCV genome transcript was then used to electroporate Huh-7.5 cells for virus production.

The sequences of *Lnc-ITM2C-1* splice variant (SP) 7, 8 and 12 RNAs (see Supplementary Table S1) and *GPR55* splice variant 1 (MANE) mRNA (see Supplementary Table S2) were overexpressed from plasmids. The expression cassette in the respective plasmids (for example, named “pAAVS_TRE3GS_Lnc-ITM2C-1SP8_HDV_IRES-EGFP”) contains, in 5′ to 3′ order, the TRE3GS promoter, the *Lnc-ITM2C-1* or *GPR55* RNA splice variant gene of interest, directly followed by a Hepatitis Delta Virus (HDV) genomic ribozyme sequence [41] to generate an authentic 3′ end of the RNA of interest, followed by the EMCV IRES element that drives translation of the coding sequence for Enhanced Green Fluorescent Protein (EGFP), in the plasmid pAAVS-iBiT-MCS-Sm-Puro plasmid (Addgene, Watertown, MA 02472, USA, #245345) [42]. Thus, a dicistronic RNA is expressed from each plasmid, with the first cistron constituted by the lncRNA or *GPR55* mRNA (cleaved off by the HDV ribozyme), and the second cistron IRES-*EGFP* as an internal reference gene. The empty vector control (“mock”) plasmid contains only the monocistronic IRES-*EGFP* cassette. The IRES-*EGFP* cassette was expressed in order to provide an equally strong “basic expression pathway load” in the cells of all four treatment groups, and also to serve as an internal reference gene for transfection and RNA transcription efficiency. In addition, these plasmids contain a human phosphoglycerate kinase (hPGK) promoter that drives expression of the reverse tetracycline-controlled transactivator (rtTA) [42]. If not described otherwise, 1 µg of these plasmids were transfected per well of a 6-well plate using Lipofectamine 2000 (Thermo Fisher Scientific, Waltham, MA 02451, USA) to Huh-7-Lunet/CD81 [43] cells 24 h prior to HCV infection in order to transiently overexpress the respective *Lnc-ITM2C-1* or *GPR55* splice variant (m)RNA.

### 2.2. Transfection

To introduce in vitro-transcribed HCV RNA genome into cells for virus production, cells were grown and washed in Opti-MEM. In total, 10 µg of HCV full-length RNA was combined with 400 µL (4 × 10^6^ cells) Huh-7.5 cell suspension. Cells were electroporated with the Gene Pulser Xcell™ (Bio-Rad, 85622 Feldkirchen, Germany) set to 270 V for 20 ms using square wave plus. Cells were resuspended in 12 mL complete DMEM, seeded in two T-175 cell culture flasks and refilled to 25 mL with complete DMEM. Cells were incubated under 5% CO_2_ at 37 °C. After 6–8 h, the old medium was changed to remove dead cells. Usually after 48 h, total RNA extraction, Western blot, or focus-forming units (FFU) assays were performed.

### 2.3. Production and Recovery of Infectious Viral Particles

To harvest infectious extracellular HCV particles from cell culture (HCVcc), virus-containing supernatants from Jc1 genome-transfected Huh-7.5 cells were harvested from 2 days up to 6 days after transfection and were stored at −80 °C until use. On day 6 after transfection, the collected supernatants were combined and thawed on ice. The thawed supernatants were centrifuged at 1000× *g* in a swing-bucket rotor for 10 min to remove cellular debris, and the supernatants were concentrated 50-fold using Amicon Ultra-15 centrifuge filter units (Merck, 64293 Darmstadt, Germany) according to the manufacturer’s instructions (3000 rpm for 20–30 min at 4 °C). The concentrated supernatant was used immediately or kept at −80 °C.

### 2.4. Infection of Cultured Cells with HCVcc

In experiments without transfection of overexpression plasmids, cells were seeded one day prior to infection. In experiments including prior transfection of overexpressing plasmids, 24 h after plasmid transfection, HCV infection was performed with a multiplicity of infection (MOI) of 0.3, while the negative control received a virus-free inoculum. Then, 4 h post infection (hpi), the medium was replaced with fresh DMEM including 10% FCS. The time points (e.g., 12, 24, 48 h) given in the results indicate cell lysis after HCV infection. For the FFU assay, infected cells were fixed, stained, and analyzed at 48 hpi.

### 2.5. Improved Total RNA Isolation and Removal of Genomic DNA

Total RNA extraction was initiated using TRIzol reagent. To prevent rapid changes in gene expression or RNA degradation, the culture medium was removed without a subsequent phosphate-buffered saline (PBS) wash. Cells were lysed with 1 mL TRIzol (Thermo Fisher Scientific, 15596026) per well of a 6-well plate for 20 min at room temperature, and the lysate was homogenized by gentle pipetting. After transfer to a 1.5 mL reaction tube, 200 µL cold chloroform was added, mixed thoroughly by gentle inversion, and incubated for 3–5 min at room temperature. Phases were separated by centrifugation (14,000 rpm, 20 min, 4 °C), and 400 µL of the aqueous RNA-containing phase was transferred to a fresh tube, very carefully avoiding transfer of the interphase. The remaining interphase and lower organic phase containing DNA and proteins were discarded.

The purity of the total RNA isolated using the above standard procedure suffices for many applications for abundantly expressed genes. However, when low abundantly expressed genes are investigated, residual and varying amounts of contaminating genomic DNA become problematic, often yielding false-positive signals in qPCR that vary from experiment to experiment. Therefore, we applied three additional steps using two or three different physicochemical principles of action for highly effective gDNA removal.

In case the resulting total RNA was used in subsequent RT-qPCR, we applied acidic phenol extraction. When phosphate backbone negative charges are largely protonated at low pH, the 2′-hydroxyl group in RNA becomes decisive for the solubility of RNA in the aqueous phase, while DNA can dissolve in the water-saturated phenol phase [44,45,46,47,48]. In total, 400 µL of acidic phenol (phenol/chloroform/isoamylalcohol, 125:24:1, pH 4.5, Thermo Fisher Scientific AM9720) were added, gently mixed by inverting the tubes 40 times, and then centrifuged at 16,000 rpm for 10 min at 4 °C. Notably, this acidic phenol performed best for gDNA removal in our hands. One likely reason is its high phenol but low chloroform content, creating an organic phase that is more hydrophilic than 50:50 mixes of phenol/chloroform, likely resulting in improved DNA solubilization in the organic phase at the low pH. However, additional features of only this reagent may also contribute to its good performance. After centrifugation, the upper aqueous phase was carefully transferred to a new tube. An equal volume of chloroform was added, the mixture was inverted 40 times and centrifuged at 16,000 rpm for 5 min at 4 °C. After transfer of the supernatant to a new tube, an equal amount of 96% ethanol was added and mixed gently but thoroughly by pipetting up and down. Thereafter, the mixture was applied to a NEB Monarch kit silica-matrix column (New England Biolabs, 65926 Frankfurt am Main, Germany, t, NEB T2040L); RNA was purified in accordance with the manufacturer’s instructions and finally eluted in 20–25 µL water. Then, the RNA preparation was subjected to recombinant “rDNase I” digestion and subsequent removal of the DNase using the “DNA-free” kit (Invitrogen/Thermo Fisher Scientific, AM1906) according to the manufacturer’s instructions.

For the preparation of cDNA to be used in NGS experiments, after standard TRIzol extraction, the total RNA was not subjected to acidic phenol extraction to avoid varying losses in RNA content. Instead, NEB Monarch columns were used directly after TRIzol, followed by two subsequent rounds of treatment with the “DNA-free” rDNase I treatment and removal kit.

### 2.6. Reverse Transcription (RT) and Quantitative Real-Time PCR (qPCR)

The RT reactions were performed with the qScript Flex cDNA Synthesis Kit (95049-100, Beverly, MA 01915, USA). The kit contains the qScript Reverse Transcriptase (RT) enzyme that also has RNase H activity. This RNase H activity results in a more differential response to different amounts of template RNA and is thus better suited for differential quantification. “RT” reactions with no reverse transcriptase enzyme added (“noRT”) were performed as negative controls to monitor residual gDNA contamination.

Initially, we used gene-specific RT primers (GSP) for detection of the HCV plus strand (NS3 region) and for detection of *Lnc-ITM2C-1* splice variant RNAs and *GPR55* and *GAPDH* mRNAs. To our surprise, such gene-specific primers often yielded false-positive RT products with the very low abundant *Lnc-ITM2C-1* and *GPR55* RNAs. Therefore, random primers were chosen for *Lnc-ITM2C-1* and *GPR55* RT reactions and achieved better results. This might appear counterintuitive at first sight; a possible advantage of random primers may be their ability to produce a huge number of diverse fragments with a broad spectrum of different sequences and high overall sequence complexity, thereby competing or suppressing strong amplification of a few mis-primed non-specific cDNA products.

For RT reactions using random primers, 0.8–1 µg of the above highly purified total RNA, 2 µL of the proprietary random primer (supplied in the QuantaBio qScript Flex cDNA Synthesis Kit, Beverly, MA, USA), and water were mixed to a final volume of 15 µL. For RT reactions using gene specific primers, total RNA, RT primer, Gene enhancer and water were mixed to a final volume of 15 µL. This mixture of RNA and primers was incubated at 65 °C for 5 min in order to denature the RNA and anneal primers. Then, the RT reaction was started by adding 1 µL of qScript Reverse Transcriptase diluted with 4 µL of the proprietary “qScript Flex Reaction Mix”. For the “noRT” control, 1 µL of water was used instead of the RT enzyme. These reactions were incubated at 25 °C for 10 min (for initial annealing and elongation of the short random primers; not for gene specific primers) and then at 42 °C for 45 min. Reactions were stopped by heating to 85 °C for 5 min and then kept at 4 °C.

Important Primer Sets (PS) for *Lnc-ITM2C-1* were PS3a (primer Lv7u-F: GGATCTGGACTCTGCCATTGTCC; Lv7u-R: CCAGGTCACATGCAAAACACGTG, amplicon 163 bp) for unique detection of splice variant (SP) 7, PS9 (Lv2/4/5/7/8ex2c-F: GGTTTTTTGACCTTGGCAATG, Lv2/4/5/7/8ex2c-R: GTGACCCTTGGTGGCTGTTTAT, amplicon 102 bp) for detection of SP2, 4, 5, 7 and 8, and PS10f (Lv12ui3-F: GCTCAGGTAGACTGACAACATAC, Lv12ui2-R: GTGGGATGAAATTGATGGTTAAGAG, amplicon 113 bp) for unique detection of SP12.

Important Primer Sets for *GPR55* mRNA were PS4 (primer Gv3/11uF: GCCTTTCTCTTTTTCTCCCCCGA, Gv3/11R: CAAAGGACACTGCCTTACCAGG) for unique detection of SP3, and based on updated transcripts in databases (2026) for SP11 (the forward primer is specific for SP3 and 11, amplicon 111 bp), PS5 (Gva-F: AAGAGAGTGAAAGGAGCCCCGTCT, Gva-R: GGGATGTGGACTGCAAACTGTAGG, amplicon 136 bp) in the common part, PS10 (Gv1/6/7/8/9ex-ex-F: CCACCAACAGTTGTGATTCAATGGGC, Gva-R: GGGATGTGGACTGCAAACTGTAGG, amplicon 230 bp) for unique detection of SP1 (the forward primer is exon-exon-spanning), PS6, 7 and 8 and other primer sets used for the differentiation of splice variants are shown in Supplementary Tables S3 and S4. *GAPDH* mRNA was detected as a positive control using primers GAPDH fwd (GAGTCAACGGATTTGGTCGT) and GAPDH rev (and RT) (GATCTCGCTCCTGGAAGATG).

qPCR reaction mixtures contained 5 µL of SsoAdvanced Universal SYBR Green Supermix (Bio-Rad), 0.3 µL each of 10 µM forward and reverse primer, 4 µL of diluted cDNA, and 0.4 µL water in a total volume of 10 µL in a 96-well PCR plate well. qPCR reactions were performed with the Bio-Rad CFX detection system with an initial denaturation at 95 °C for 30 s, followed by 39 cycles of denaturation at 95 °C for 3 s and primer annealing and extension at 60–62 °C (depending on primer pairs) for 30 s. Subsequently, a melting curve was generated by heating from 65 to 95 °C with a temperature increment of 0.5 °C every 5 s.

### 2.7. Western Blots and Immunofluorescence

For protein analyses by Western Blot (WB), cells were washed twice with ice-cold PBS and lysed with RIPA buffer (50 mM Tris-HCl pH 8.0, 150 mM NaCl, 1% Triton X-100, 0.5% Sodium Deoxycholate, 0.1% Sodium Dodecyl Sulphate (SDS) and 1 mM NaF, containing protease inhibitors cOmplete™, Mini, Protease Inhibitor Cocktail, EDTA-free in 10 mL, #04693159001, Roche, 79639 Grenzach-Wyhlen, Germany) for 15 min with constant swirling. The cell lysates were mixed thoroughly by pipetting, collected into 1.5 mL tubes and incubated for another 15 min on ice. To remove cell debris, the cell lysate was centrifuged at 14,000 rpm for 15 min at 4 °C. Then, the supernatant was transferred to a fresh tube and kept on ice for further processing or stored at −40 °C.

Proteins were resolved by SDS Polyacrylamide Electrophoresis (SDS-PAGE). Western Blots were performed to analyze HCV NS3 protein expression, using GAPDH as a loading control. Before staining with a primary antibody, the membrane was incubated with WB blocking solution (5% milk powder in PBS with 0.3% Tween) overnight with gentle agitation on a rocking shaker at 4 °C. Primary antibodies were directed against HCV NS3 protein (Abcam, Cambridge, UK, CB2 0AX, UK, AB13830, RRID:AB_300673, 1:1000 for WB and 1:500 for IF) and GAPDH (Clone 6C5, Sigma-Aldrich CB1001, RRID:AB_2107426, 1:10,000 for WB, Merck, 64293 Darmstadt, Germany). After the protein gel run and transfer to nitrocellulose membrane, the membrane was horizontally cut to allow specific staining with two different primary antibodies. The primary antibodies were diluted in 10 mL PBS with 0.1% Tween (PBST) and 0.5% milk powder (WB blocking solution) and incubated for 1.5 h at room temperature with gentle agitation. After incubation, the membrane was washed three times with PBST for 10 min each, followed by incubation with the secondary anti-mouse-IgG peroxidase-coupled antibody (Sigma-Aldrich A9044, RRID:AB_258431) diluted 1:1000 in WB blocking solution for 45 min at room temperature with gentle agitation. The membrane was then washed three times with PBST and incubated with the developing solution (Amersham ECL Select Western Blotting Detection) for 5 min, followed by detection and visualization with the Bio-Rad ChemiDoc system.

For immunofluorescence (IF) staining, the supernatant medium was removed, and cells were washed slowly with cold PBS. Cell fixation was performed with 4% paraformaldehyde (PFA) at room temperature for 30 min. Cells were then washed once with PBS and permeabilized with PBS with 0.1% Tween for 5 min at room temperature, slowly washed three times with PBS and incubated for 1 h with IF blocking solution (10% goat serum and 1% BSA in PBS) at room temperature. The primary antibody (anti-HCV NS3, Abcam AB13830) was diluted 1:500 in IF blocking solution and incubated for 1.5 h at room temperature. Subsequently, the cells were washed three times in ice-cold PBST and stained with Goat anti-Mouse IgG1 Cross-Adsorbed Secondary Antibody (Alexa Fluor™ 488, Invitrogen/Thermo Fisher Scientific, A-21121) after 1:250 dilution in IF blocking solution, together with 1 µg/ml DAPI nuclear stain, in the dark for 1 h. Thereafter, cells were washed three times with PBS with 0.01% Tween, and imaging was performed with a BZ-X810 compact fluorescence microscope operated by the BZ-X800 software, Version 1.3.1.1; IF images were taken with a 20 × Plan Fluor objective (NA 0.45, (Keyence Corporation, Osaka, Japan)).

### 2.8. cDNA Library Preparation and Next-Generation Sequencing (NGS)

Two experimental combined groups of Huh-7-Lunet/CD81 cells were established for the NGS experiment: one combined group was HCV-infected at an MOI of 0.3, while the other combined group received a similar but virus-free inoculum. Within each combined group, each of the four treatment groups were transfected 24 h prior to HCV-infection with 1 µg of plasmid per well of a 6-well plate for overexpression of either *Lnc-ITM2C-1* splice variant 8 (SP8), *Lnc-ITM2C-1* splice variant 12 (SP12), *GPR55* mRNA splice variant 1, or no expression cassette insert, i.e., with plasmids:

pAAVS_TRE3GS_Lnc-ITM2C-1SP8_HDV_IRES-EGFP

pAAVS_TRE3GS_Lnc-ITM2C-1SP12_HDV_IRES-EGFP

pAAVS_TRE3GS_GPR55_HDV_IRES-EGFP

pAAVS_TRE3GS_IRES-EGFP (“mock”).

Accordingly, two RNAs are released from the dicistronic RNA by the HDV ribozyme, first the lncRNA or *GPR55* (m)RNA, and second the IRES-*EGFP* RNA. Expression of these genes of interest in the Huh-7-Lunet/CD81 cells was driven by the plasmid-encoded TRE3GS promoter. The plasmids additionally encoded the rtTA transactivator under the control of the hPGK promoter, allowing doxycycline-dependent induction without requiring stable expression of rtTA in the host cells.

The empty vector negative control (“mock”) plasmid expresses only the monocistronic IRES-EGFP RNA. The IRES–*EGFP* cassette was expressed to provide a comparable basal expression burden in the cells of all four treatment groups; however, *EGFP* expression was not used as the principal readout in the present study. Consistently, *EGFP* transcript levels did not differ significantly among the treatment groups at 48 hpi. The entire experiment was repeated in three biological replicates (R1, R2 and R3) which were later combined for the secondary bioinformatic data analysis.

rRNA depletion was chosen instead of poly(A)-based enrichment to obtain a broader representation of the transcriptome including lncRNAs, while reducing rRNA content before RNA-Seq. Depletion was performed using the NEBNext^®^ rRNA Depletion Kit v2 (Human/Mouse/Rat), according to the manufacturer’s instructions, to provide better coverage of non-ribosomal transcripts. Quality and quantity of the libraries were assessed with the Agilent 4200 TapeStation and D5000 ScreenTape (Agilent, 76337 Waldbronn, Germany). Finally, all libraries were pooled, and sequencing was performed on an Illumina NovaSeq 6000 instrument (San Diego, CA 92122 USA) (using S1 300 cycles v1.5) configured for paired-end mode with 2 × 150 bp reads. The paired-end sequencing provides sequence information from both ends of each library fragment and improves alignment accuracy as well as transcript quantification.

### 2.9. Bioinformatic Data Analysis

The Illumina paired-end sequencing data contained Unique Molecular Identifiers (UMIs), which were used to reduce potential amplification bias in downstream quantitative analyses.

Briefly, the automated workflow comprised quality filtering and adapter trimming using fastp (v. 0.23.4) [49], sequencing quality control was performed using FastQC (v. 0.12.1) (https://www.bioinformatics.babraham.ac.uk/projects/fastqc/, accessed 10/09/2026) and summarized across samples using MultiQC (v. 1.33) [50]. This was followed by alignment to the T2T-CHM12v2.0 reference genome (GCF_009914755.1) using HISAT2 (v. 2.2.2) [51]. SAM files were converted to BAM format and indexed using SAMtools (v. 1.23) [52]. PCR duplicates were subsequently removed based on the UMIs using UMI-tools (v. 1.1.6) (https://umi-tools.readthedocs.io/en/latest/, accessed 10/09/2026). Read counting was performed with featureCounts (v. 2.1.1) [53] in paired-end mode, using exon features (-t exon) grouped by gene_id (-g gene_id) and the gene_id attribute provided in the GTF annotation. The annotation file used was GCF_000001405.40_GRCh38.p14_genomic.gtf, corresponding to the GRCh38.p14 human genome assembly. No major difficulties were encountered at the read-alignment level, although interpretation of previously reported Lnc-ITM2C-1 transcripts required careful comparison with the updated annotation, due to modification of genomic boundaries and transcript models between different databases. Features corresponding to known rRNAs were removed from the resulting count table to minimize bias caused by their overrepresentation.

The resulting count data were imported into R for downstream statistical analysis using DESeq2 (v. 1.50.2) [54]. Variance-stabilizing transformation was applied to the count data for visualization. The R workflow was used to generate principal component analysis (PCA) plots, volcano plots, and differential expression tables.

### 2.10. Other Data Processing

The adjusted *p*-values (*p*adj) and log2-fold changes shown in the volcano plots were calculated using DESeq2 (v. 1.50.2) [54]. Other data analysis and display (that is not direct DESeq2 output) was performed using Microsoft Excel and GraphPad Prism software (version 8.4.3 for Windows, GraphPad Software, San Diego, CA, USA, www.graphpad.com) using RT-qPCR analyses or the read count output from DESeq2. For example, this includes the calculation of log_2_ (curated read counts) shown in the violin plots and in the histograms. Unnormalized read counts were used for the violin plots, normalized read counts for most histograms. Multiple T significance or two-way ANOVA tests were calculated in GraphPad. *n*: number of biological replicates; significances: * (*p* < 0.05), ** (*p* < 0.01), *** (*p* < 0.001), **** (*p* < 0.0001), and ***** (*p* < 0.00001); n.s. = not significant (*p* > 0.05).

Once it became evident that the overexpression of *Lnc-ITM2C-1* or *GPR55* mRNA had no significant effects on the differential expression of any gene (except the slight downregulation of one gene, *RNU5B1*, under overexpression of *Lnc-ITM2C-1* splice variant 8), we combined the four lncRNA or *GPR55* overexpression treatments, each with three biological replicates. Thus, 12 uninfected and 12 HCV-infected samples were used to recalculate a revised version of the volcano plot showing the effect of HCV infection on differential gene expression.

## 3. Results

### 3.1. Improved Removal of gDNA Is Essential for Reliable Analysis of Low-Abundance Genes

During our investigation of *Lnc-ITM2C-1* and *GPR55* RNAs, contamination of total RNA preparations with genomic DNA (gDNA) became increasingly problematic. Motivated by this observation, by reports of gDNA-contamination issues [38] and by the advantages of improved RNA purification in an HCV-replication study [48], we aimed to improve the reliability of RT-qPCR analyses by substantially enhancing gDNA removal from total RNA preparations. Therefore, in comparison with the standard TRIzol RNA purification procedure, we added three steps to improve removal of residual gDNA.

**Figure 1:**
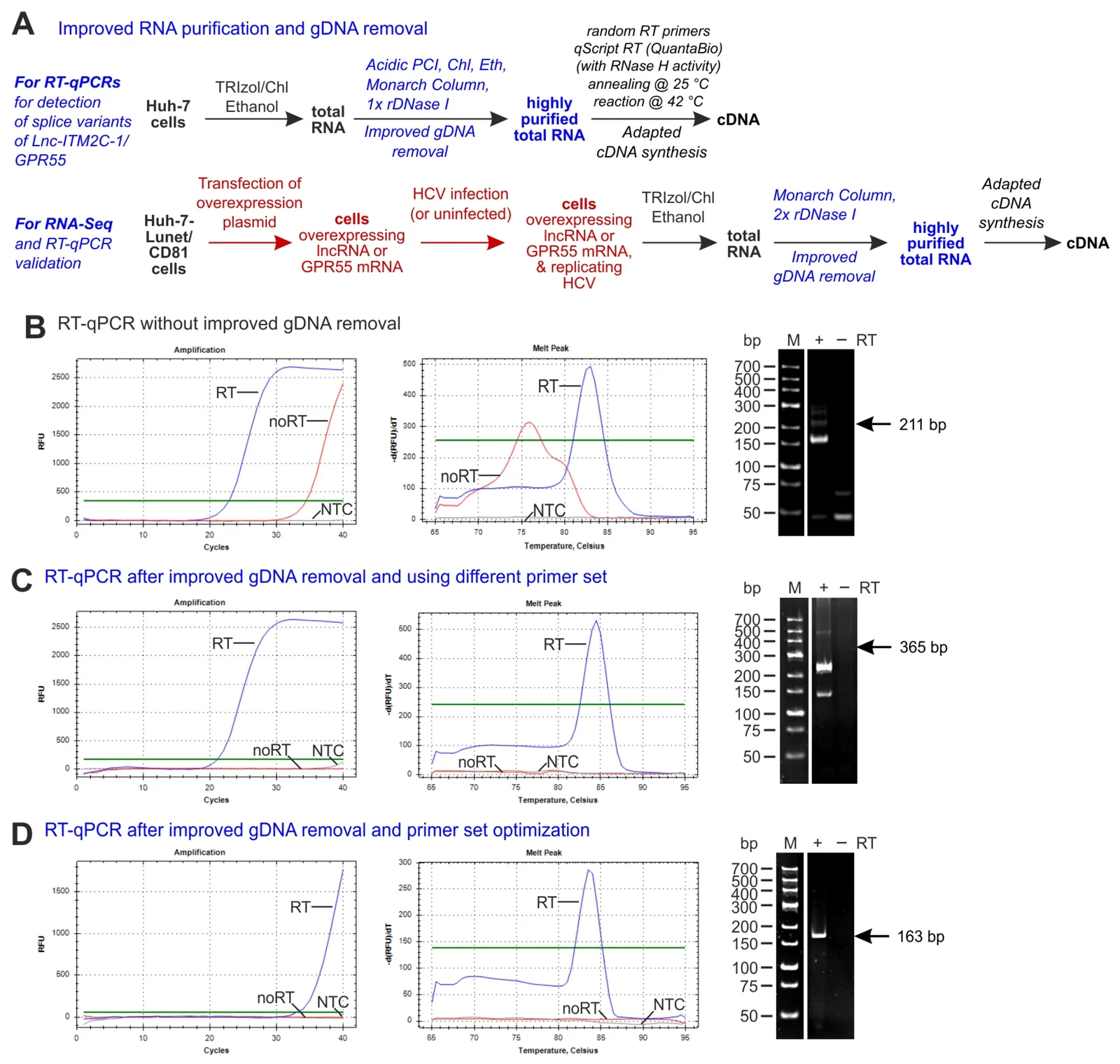
Improvement of RNA purification for RNA-Seq and RT-qPCR analyses. (**A**) Protocols for improved RNA purification, removal of gDNA and cDNA preparation. Top: protocol for preparation of total RNA for use in RT-qPCRs, bottom: protocol for preparation of total RNA for use in RNA-seq and directly related RT-qPCR validations. Transfections and HCV infections for the RNA-seq experiment are shown in red, and improved RNA purification procedures are in blue. (**B**–**D**) Characterization of RT-qPCR quality and gDNA removal. (**B**) RT-qPCR result following standard TRIzol RNA purification procedures. The left panel shows qPCR amplification Ct curves for the RT reaction including the Reverse Transcriptase (RT) enzyme, a control reaction without added RT enzyme (noRT), and a control containing no template (NTC). The middle panel shows the corresponding melt curves, and the right panel shows an acrylamide gel with the RT-qPCR products. In this case, amplification was detected in the noRT negative control, and the noRT melt curve showed a broad peak with shoulders. In the gel, an unspecific strong qPCR product band appeared, and a very faint band of the expected size, together indicating multiple and largely unspecific (mis)priming. Accordingly, this primer set (PS1a for *Lnc-ITM2C-1*, see Supplementary Materials) was not further used. (**C**) RT-qPCR product analysis after using the improved gDNA removal protocol developed in this study and using another primer set (PS1). The noRT shows no amplification curve and the melt curve shows one peak. In the gel, multiple products with one major and two minor bands of wrong sizes appeared, whereas a product of the correct size is not detected. Thus, this primer set was not further used. (**D**) RT-qPCR analysis after improved gDNA removal, combined with an optimized and validated primer set (PS3a for *Lnc-ITM2C-1*). The RT Ct curve shows a higher Ct, consistent with optimized detection of a low abundantly expressed gene, and on the gel, only one product band of the correct size is visible. Uncropped gels are shown in Supplementary Figure S1A–C, Ct values are shown in Supplementary Figure S1D.

These steps included the removal of DNA by acidic phenol extraction in cases when RNA was subsequently used for individual target validation RT-qPCR analyses. This step was omitted when preparing RNA for NGS, since acidic phenol extraction could cause unwanted variations in total RNA recovery in our hands. In all cases, a silica matrix-based column purification of RNA (here: NEB Monarch columns) was used for the procedure. This step was followed by either one (with RNA used for individual target validation RT-qPCR) or two (with RNA used for NGS) subsequent rounds of recombinant rDNase I treatment and removal. Thus, in comparison to standard TRIzol-based RNA purification, three additional steps with at least two different physicochemical principles of action were used to thoroughly remove residual gDNA from our total RNA preparations (Figure 1A).

The success of this improved gDNA removal—in combination with primer optimization and qPCR product validation steps—is shown in Figure 1B–D. Standard TRIzol RNA purification yielded a qPCR amplification curve with a Ct value of 23 for the detection of *Lnc-ITM2C-1* RNA (Figure 1B, left panel, “RT”). This qPCR also resulted in a melting curve with a single peak (middle panel, “RT”).

However, when we omitted the Reverse Transcriptase enzyme in the “noRT” control, the resulting amplification with a Ct value of 34 (left panel) and a broad melt curve with pronounced shoulders (middle panel) indicated gDNA contamination and other non-specific products, e.g., primer dimers. As an additional control, we then loaded the qPCR products on a non-denaturing polyacrylamide gel (right panel). The RT-qPCR reaction with the sharp melt curve had yielded a major product band, but to our surprise, that major band had the wrong size, while one of the three faint additional bands had the correct size. In the noRT control, two smaller fragments are visible. Thus, despite seemingly flawless amplification and melt curve with a sharp peak, the RT-qPCR reaction was not successful but produced unspecific false-positive signals. Moreover, for such a low-abundance expressed gene, a Ct of 20 was not expected. After improved gDNA removal and choice of a new primer set (Figure 1C), the noRT control was negative in the Ct and melting curves as well as in the gel. However, multiple unspecific RT-qPCR products appeared in the gel. This improvement might be caused by using an optimized primer set or by the improved gDNA removal method. The only aim to represent this result is to demonstrate that flawless amplification and melting curves and a negative noRT control do not suffice to validate product specificity. Only after choosing another primer set with a smaller amplicon length and optimized primer specificity (Figure 1D), the gel showed a strong RT-qPCR product band of the correct size, while the noRT control was negative. Remarkably, the Ct value of the RT-qPCR reaction was high, which—in combination with the single amplification product in the gel—is consistent with an increased specificity of the RT-qPCR reaction as well as with the suspected low abundance of the lncRNA transcript (Ct values for the above experiments are shown in Supplementary Figure S1D). Eventually, we checked the outcomes of the two RNA purification strategies for RT-qPCR analyses and for RNA-Seq to be the same with respect to the Ct curves, melt curves and negative noRT controls (Supplementary Figure S1E).

These results highlight the importance of validating RT-qPCR assays for low-abundance expressed genes by using noRT controls and gel-based verification of the expected product size. These measures enable assessment of residual gDNA contamination and allow validation of primer sets for specific detection of low-abundance transcripts.

In the following, primer sets for the successful detection of *Lnc-ITM2C-1* and *GPR55* transcripts in Huh-7 cells were validated as above. gDNA contamination was evaluated by comparing positive RT with noRT reactions. For low-abundance targets close to the detection limit, only primer sets that showed no increase up to a Ct value of 40 in the noRT controls were used. For high-abundance transcripts, noRT signals occurring at least 10 Ct values later than the RT signals were considered unlikely to substantially affect quantification.

### 3.2. Expression of Lnc-ITM2C-1 and GPR55 mRNA Splice Variants in Huh-7 Hepatoma Cells

After the above optimizations, we analyzed the expression of splice variants of *Lnc-ITM2C-1* RNA and *GPR55* mRNA that were database-annotated at that time, in order to find out which of these splice variants are expressed in Huh-7 cells and might be relevant for HCV replication.

**Figure 2:**
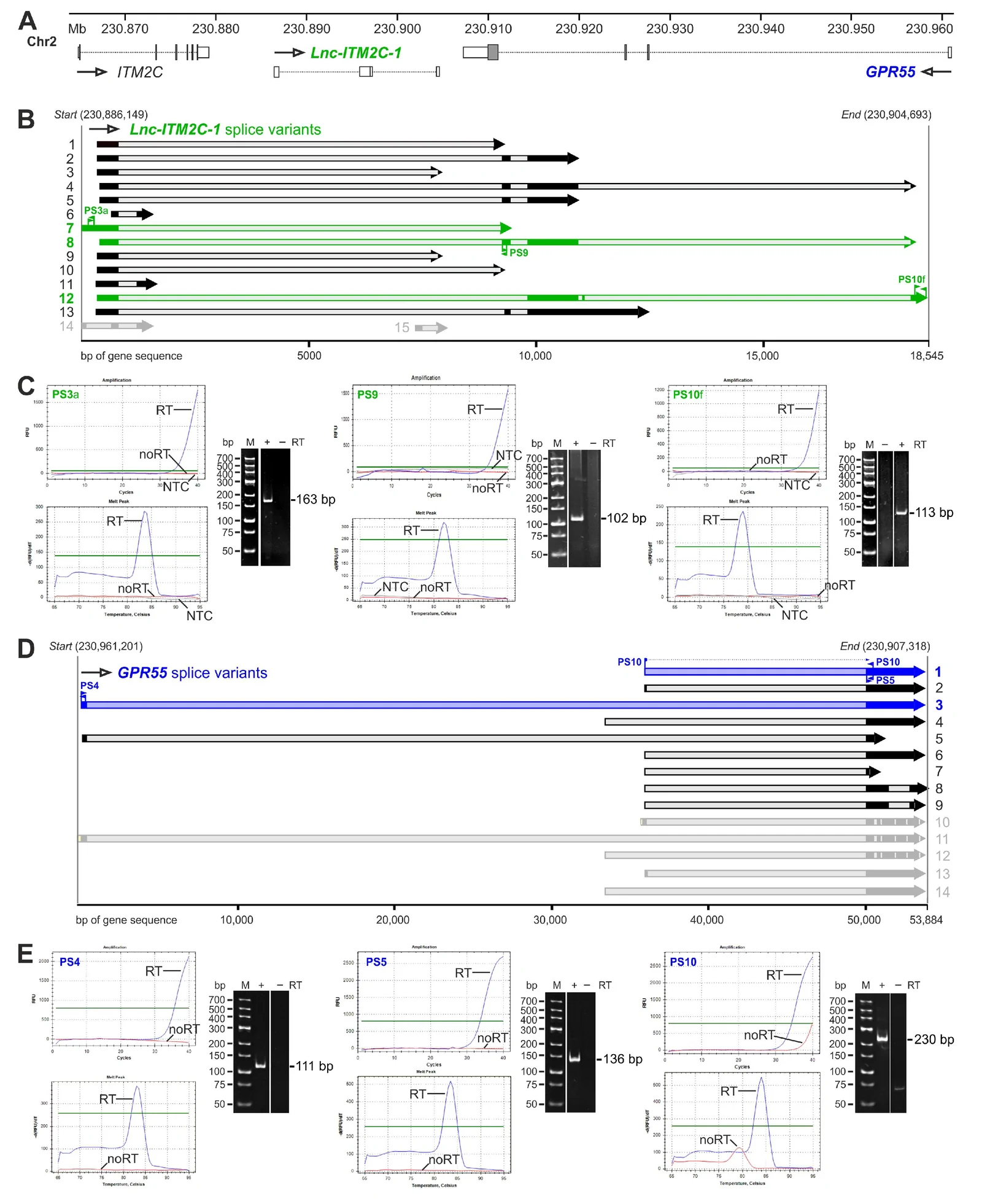
*Lnc-ITM2C-1* and *GPR55* genes and splice variants. (**A**) The genomic region with the protein coding genes *ITM2C* and *GPR55* and the lncRNA *Lnc-ITM2C-1*. Arrows indicate the direction of transcription; *GPR55* is transcribed from right to left in panel A. Redrawn based on information from: https://www.ensembl.org/Homo_sapiens/Location/View?db=core;g=ENSG00000232520 (accessed on 25 June 2026), Location: 2:230,860,000–230,965,000, encompassing the *ITM2C* and *GPR55* genes. Non-coding exons are drawn as white boxes, protein coding sequences as gray boxes. (**B**) Splice variants known for *Lnc-ITM2C-1*. Here, exons are drawn in dark (black or green), introns in light gray. Splice variants found expressed in Huh-7 cells and derivatives in this study are in green. Primer sets (PS) 3a, 9 and 10f were used for detection of *Lnc-ITM2C-1* splice variants by RT-qPCR, forward and reverse primers are indicated by flags. PS3a unambiguously identified expression of SP7. PS10f unambiguously identified expression of SP12. SP8 was detected by PS9 targeting a region shared in SP2, 4, 5, 7 and 8; however, SP8 is the most likely splice variant according to additional information. Splice variants in *Lnc-ITM2C-1* (**B**) and *GPR55* (**D**) not yet known at the beginning of this study are in gray. (**C**) RT-qPCR analysis (like in Figure 1D) of SP7, 8 and 12 by the above primer sets (uncropped gels are shown in Supplementary Figure S2A–C). The results for PS3a are the same as shown in Figure 1D. (**D**) Splice variants known for the *GPR55* gene. Exons are shown in black (coding sequences are not specifically marked here), introns in gray. Splice variants found in this study to be expressed in Huh-7 cells and derivatives are in blue. (**E**) RT-qPCR analysis of *GPR55* SP1 and 3 by the above primer sets. PS4 targets a sequence unique to splice variant 3. PS10 detects SP1 (and SP6–9) by an exon–exon junction forward primer, while database information and negative results using primer sets specific to other splice variants argued for SP1. Validation of GPR55 expression in Huh-7 cells was confirmed by using PS5, targeting the common region of all transcripts. Uncropped gels are shown in Supplementary Figure S3A–C. Another RT-qPCR analysis using the PS10 primer set supports specificity by a single well-defined melting peak and negative noRT and NTC controls (please see Supplementary Figure S3D). However, acrylamide gel electrophoresis was not performed for these samples, i.e., the amplicon size was not independently verified. Ct values are shown in Supplementary Figure S2D.

Despite their low reported abundance in liver expression datasets, *Lnc-ITM2C-1* and *GPR55* splice variants were investigated in Huh-7 cells because the transcript had previously been detected in this HCV-relevant context, and individual low-abundance splice variants may not have been reliably detected in different databases. Using primer sets (PS) that each are specific for a given splice variant, we unambiguously identified expression of splice variants (SP) 7 (with PS3a) and 12 (with PS10f) of *Lnc-ITM2C-1* (Figure 2A–C). In contrast, identification of SP8 remained ambiguous, although SP8 is the reference sequence in NCBI that is regarded as representative of the biology at that locus. Unfortunately, SP8 has no sequences that are unique to it. Therefore we considered it to be likely expressed, mainly based on the fact that it is the representative NCBI RefSeq variant, but also due to some indirect experimental evidence from amplification with primer sets targeting sequences shared with SP8 and other splice variants. Taken together, the *Lnc-ITM2C-1* locus expresses SP7 and SP12, and likely the NCBI RefSeq transcript SP8 as splice variants in Huh-7 hepatoma cells. Nevertheless, since SP8 was introduced as the RefSeq transcript in NCBI, we preferred to overexpress SP8 instead of SP7 in the following RNA-Seq experiments.

Accordingly, we found *GPR55* SP1 and SP3 expressed in Huh-7 cells (Figure 2D,E). SP3 was identified by primer set PS4 that is unique to the 5′ end of SP3. Here, by using primer set PS4, the upstream sequence extension of SP3 that is annotated in NCBI but not yet in EMBL-EBI was confirmed. SP1 was identified by the exon–exon junction forward primer in PS10 (which can also detect SP6–9) and by database information.

### 3.3. Genes Specifically Upregulated by HCV Infection

Then, we aimed at an overall analysis of differential gene expression in HCV-infected cells. In this context, we also wanted to extend our previous study, which had suggested that HCV induces *Lnc-ITM2C-1* expression, and *Lnc-ITM2C-1* may indirectly favor HCV replication by supporting expression of its neighboring gene *GPR55*, which in turn may suppress selected ISGs [37]. Our refinement is aimed at analyzing which splice variants of the respective genes are upregulated, and which effect the expression of each of these splice variants may have. Therefore, we prepared a panel of 24 samples in total, of which 12 samples were assigned to the combined HCV-infected group, and 12 samples were assigned to the combined uninfected group. Within each combined group, four treatment groups with each three biological replicates were prepared. These treatment groups overexpressed either *Lnc-ITM2C-1* SP8, SP12, *GPR55* SP1, or empty vector control (“mock”). For that, plasmids were transfected into the cells 24 h prior to HCV infection to transiently overexpress the respective genes. As cells we used Huh-7-Lunet/CD81 cells [43] that overexpress one of the HCV receptors, the CD81 protein, to facilitate HCV entry.

For selecting the desired time point for the samples sent for RNAseq, we first checked successful infection at different MOIs (Supplementary Figure S4A) of the Huh-7-Lunet/CD81 cells. An MOI of 0.3 was chosen since this MOI was sufficient to yield good virus production, even though the fraction of infected NS3-positive cells was about 20–28% of all DAPI-positive cells with that MOI (Supplementary Figure S4B). Moreover, we used an MOI of 0.3 also in our previous paper [37], and therefore we considered making this study comparable to the previous one regarding the context of infection. Supplementary Figure S4C shows a time course experiment. The 48 hpi time point was chosen for further experiments, since it is a time point with fully established and robust replication of HCV [48,55]. For plasmid transfections, 1 µg per well of a 6-well plate was chosen, since this resulted in a good balance between gene expression and cell viability (Supplementary Figure S4D,E). Endogenous Lnc-ITM2C-1 SP8 was detected by RT-qPCR with a Ct value of about 33 in uninfected and HCV-infected cells (Supplementary Figure S4F, first two values “Mock” and “HCV-Mock”), whereas overexpression of the *Lnc-ITM2C-1* or *GPR55* RNAs resulted in their overexpression by about 7 to 9 log_2_ steps (i.e., 128- to 512-fold). In contrast, expression of the cellular reference genes *β-actin* and *GAPDH* as well as the EFGP reference gene expressed from the transfected plasmids remained unchanged (Supplementary Figure S4G–I). Since the low-level leaky activity of the TRE3GS promoter driving Lnc-ITM2C-1 or GPR55 expression from the plasmid was sufficient to raise expression levels by 7 to 9 log_2_-steps over their endogenous expression (Supplementary Figure S4F,J,K), and doxycycline further raised expression by about 3 to 4 log_2_-steps in our hands, we decided to omit doxycycline induction to avoid raising lncRNA levels to perhaps unphysiologically high concentrations, and also avoid possible doxycycline side effects on gene expression and metabolism [56,57].

**Figure 3:**
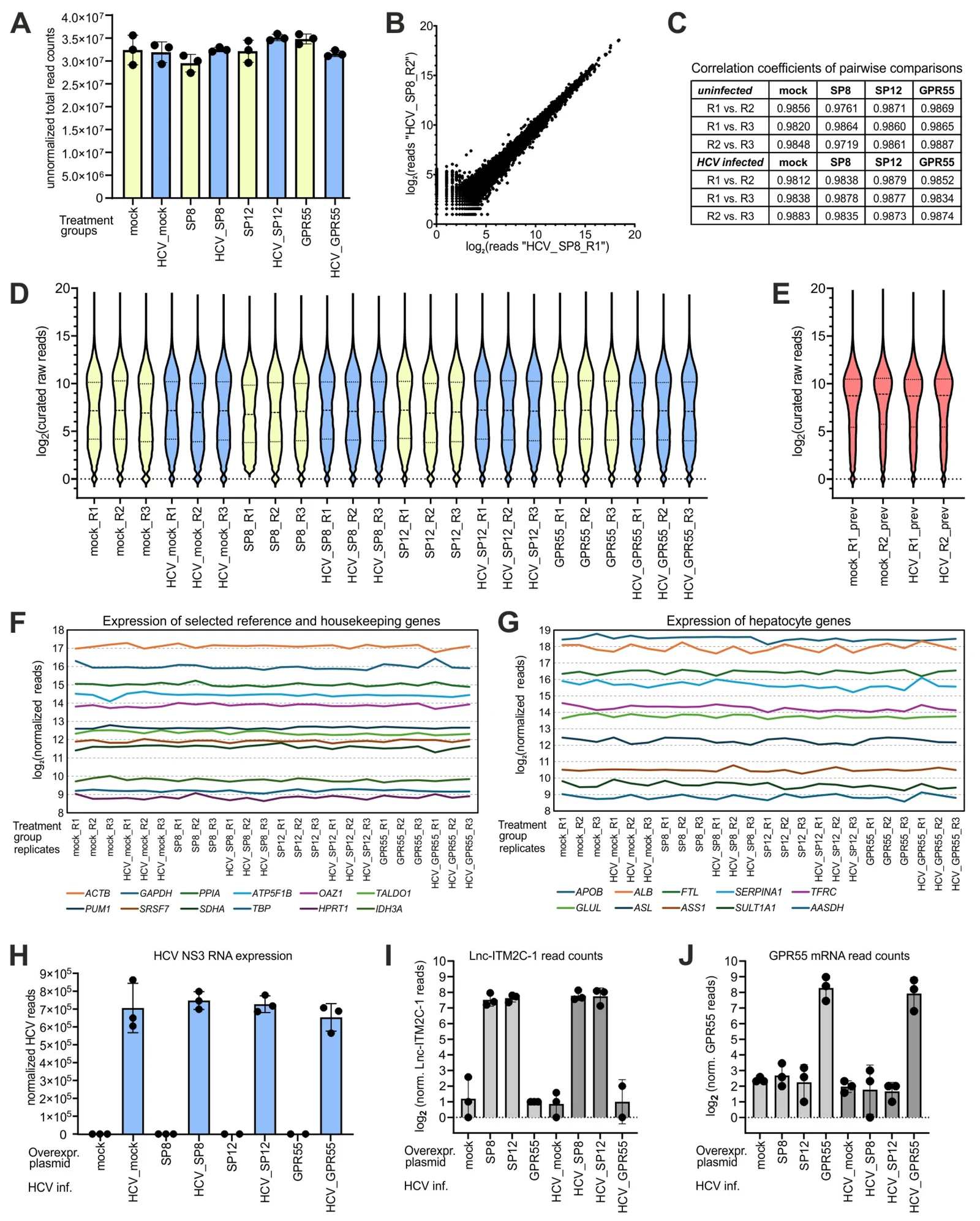
Quality assessment and consistency of RNA-Seq data. (**A**) Unnormalized total curated read counts (means and SDs) of the four treatment groups (overexpression of *Lnc-ITM2C-1* splice variant 8 (“SP8”), or “SP12”, or “*GPR55*”, or empty plasmid = “mock”), each including three replicates (black dots), in the combined groups “no infection” (light yellow) or “HCV infection” (blue). (**B**) One example of pairwise comparison of log_2_ (normalized read counts) among the three replicates of each treatment group; each dot represents log_2_ (normalized read counts) of a specific gene in one replicate vs. the log_2_ (normalized read counts) of the same gene in the other replicate. All pairwise comparisons are shown in Supplementary Figure S5. (**C**) shows the corresponding correlation coefficients of all pairwise comparisons within the treatment groups (from Supplementary Figure S5). In the Supplementary Materials, the data analysis quality report (multiqc_report) is also provided. (**D**) Violin plots of the distribution of log_2_ (unnormalized read counts) of all 24 NGS treatment group replicate (R) samples. In (**D**,**E**), big dotted lines indicate the median, small dotted lines quartiles. (**E**) For comparison, violin plots of 4 samples from a different RNA-Seq experiment without improved gDNA removal (not further used in this study). (**F**) Detection of a panel of recommended reference genes in all replicates, not including ribosomal RNAs. (**G**) Detection of some hepatocyte genes. (**H**) HCV genome-specific normalized read counts in all samples, confirming HCV RNA replication in HCV-infected samples. (**I**) Log_2_ (read counts) of *Lnc-ITM2C-1* in all RNA-Seq samples. Here, “mock” indicates that a plasmid without gene of interest (GOI) was transfected. (**J**) Log_2_ (read counts) of *GPR55* mRNA in all RNA-Seq samples.

Quality and reproducibility of the NGS data were checked by analyzing total sample read counts (Figure 3A), pairwise replicate comparisons (Figure 3B,C and Figure S5), “violin plots” of the read count distribution in all replicates of the experiment used in this study (Figure 3D) and, for comparison, violin plots of the read count distribution from our previous RNA-Sequence experiment (from 2021) not further used in this study (Figure 3E), expression of a panel of 12 different recommended reference genes [58,59,60,61] (Figure 3F) and 10 hepatocyte relevant genes [62] (Figure 3G), HCV read counts (Figure 3H), as well as read counts of endogenous and overexpressed *Lnc-ITM2C-1* splice variant RNAs (Figure 3I) and *GPR55* mRNA (Figure 3J). All results showed reproducibility for all tested parameters across all replicates. In particular, the read count distribution in Figure 3D shows reliable distribution also of low-abundant reads, consistent with a low contamination of the samples with gDNA [40]. From the comparison of Figure 3D with Figure 3A, we can estimate that HCV genome reads constituted about 2% of the total read counts of the sample.

**Figure 4:**
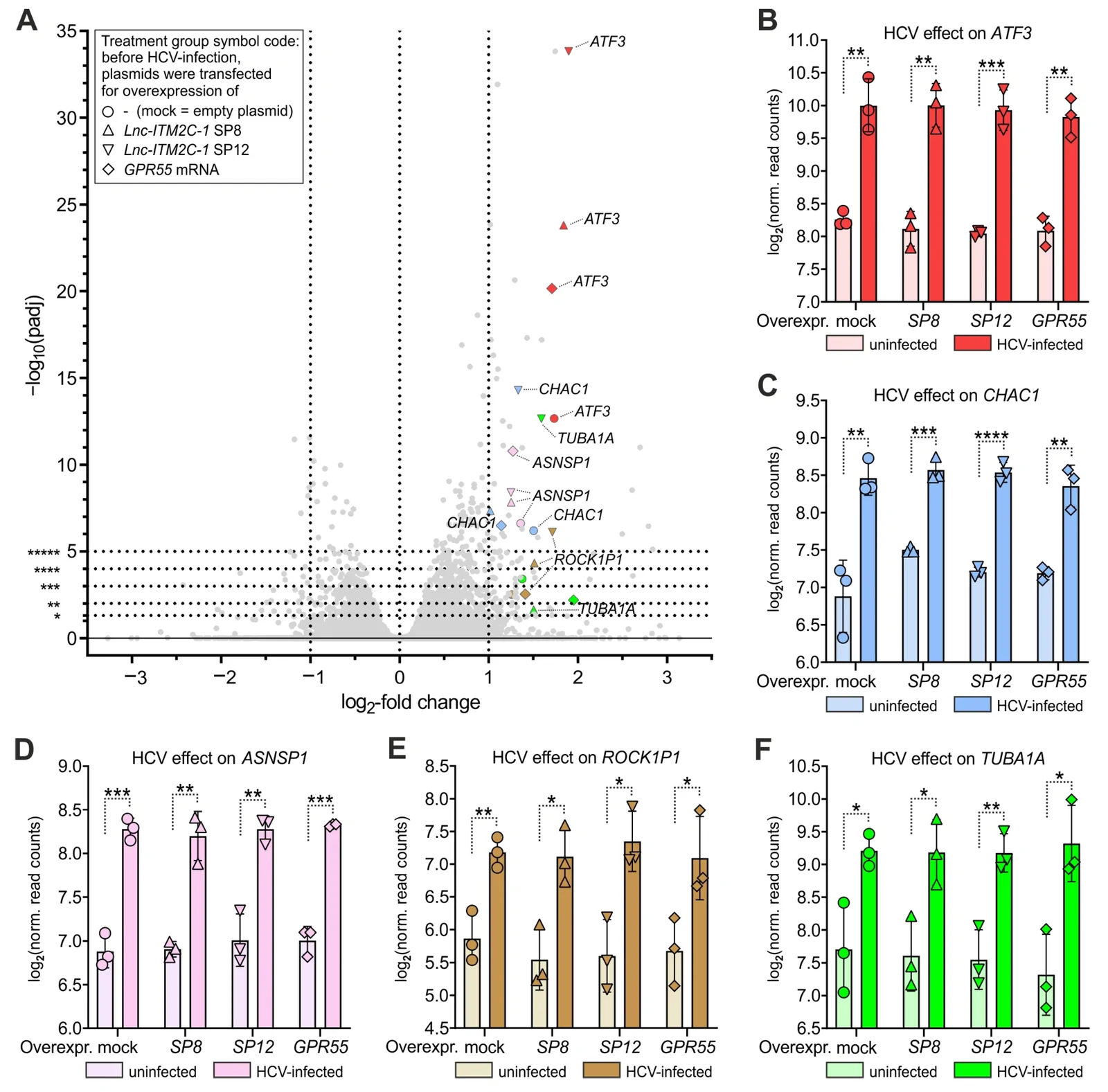
NGS analysis of the effect of HCV infection on gene expression in Huh-7 Lunet/CD81 cells, pre-transfected in four treatment groups with plasmids overexpressing *Lnc-ITM2C-1* SP8, SP12, *GPR55* mRNA SP1, or empty plasmid (“mock”). (**A**) Volcano plot of differential gene expression after HCV infection. Symbols show −log_10_(*p*adj) values over log_2_-fold change for the effect of HCV infection. Asterisks indicate adjusted *p*-values with * <0.05, ** <0.01, *** <0.001, **** <0.0001 and ***** <0.00001. The symbol color specifies a specific target gene, the symbol shape specifies the overexpression treatment group. Only those genes that showed *p*adj-values of less than 0.05 (*) and a log_2_-fold change > 1 in each of the four treatment groups are particularly labeled and also detailed in panels (**B**–**F**), which show the log_2_ (normalized read counts) of HCV-infected vs. uninfected samples separately for the four overexpression treatment groups (with the same symbol code as in (**A**); e.g., “*ATF3*-*GPR55*” data are shown using a red diamond). Here, “mock” indicates that a plasmid without GOI was transfected. Asterisks indicate (not adjusted) *p*-values from *t*-tests of pairwise comparisons with * <0.05, ** <0.01, *** <0.001, **** <0.0001 and ***** <0.00001.

After HCV infection, expression of several genes is significantly upregulated, while nearly no genes are significantly downregulated (Figure 4A). Only those genes that are upregulated during HCV infection in all four overexpression treatment groups are marked in the volcano plot. The respective genes are similarly upregulated during HCV infection under all overexpression treatments, after overexpression of *Lnc-ITM2C-1* SP8 and SP12 as well as *GPR55* mRNA SP1, and even with no overexpression (“mock”) (detailed in Figure 4B–F).

The gene that is most prominently upregulated under all four treatments is *ATF3* (Activating Transcription Factor 3) which was reported previously to be involved in the stress response during HCV infection [63,64,65,66]. *CHAC1* (Glutathione Specific γ-Glutamylcyclotransferase 1) is also involved in stress response [67,68], and the other genes found upregulated are *TUBA1A* (tubulin 1α) and two pseudogenes, *ASNSP1* and *ROCK1P1*. The expression of four of the five genes detected to be differentially expressed during HCV replication (except the pseudogene *ROCK1P1*) are moderately or strongly expressed, with log_2_ values of read counts between 7 and 10 (compare Figure 3D). Notably, under all treatment conditions of *Lnc-ITM2C-1* RNA or *GPR55* mRNA overexpression, the response of the above genes to HCV infection is very similar, and the overexpression treatment did not alter the response to HCV infection.

**Figure 5:**
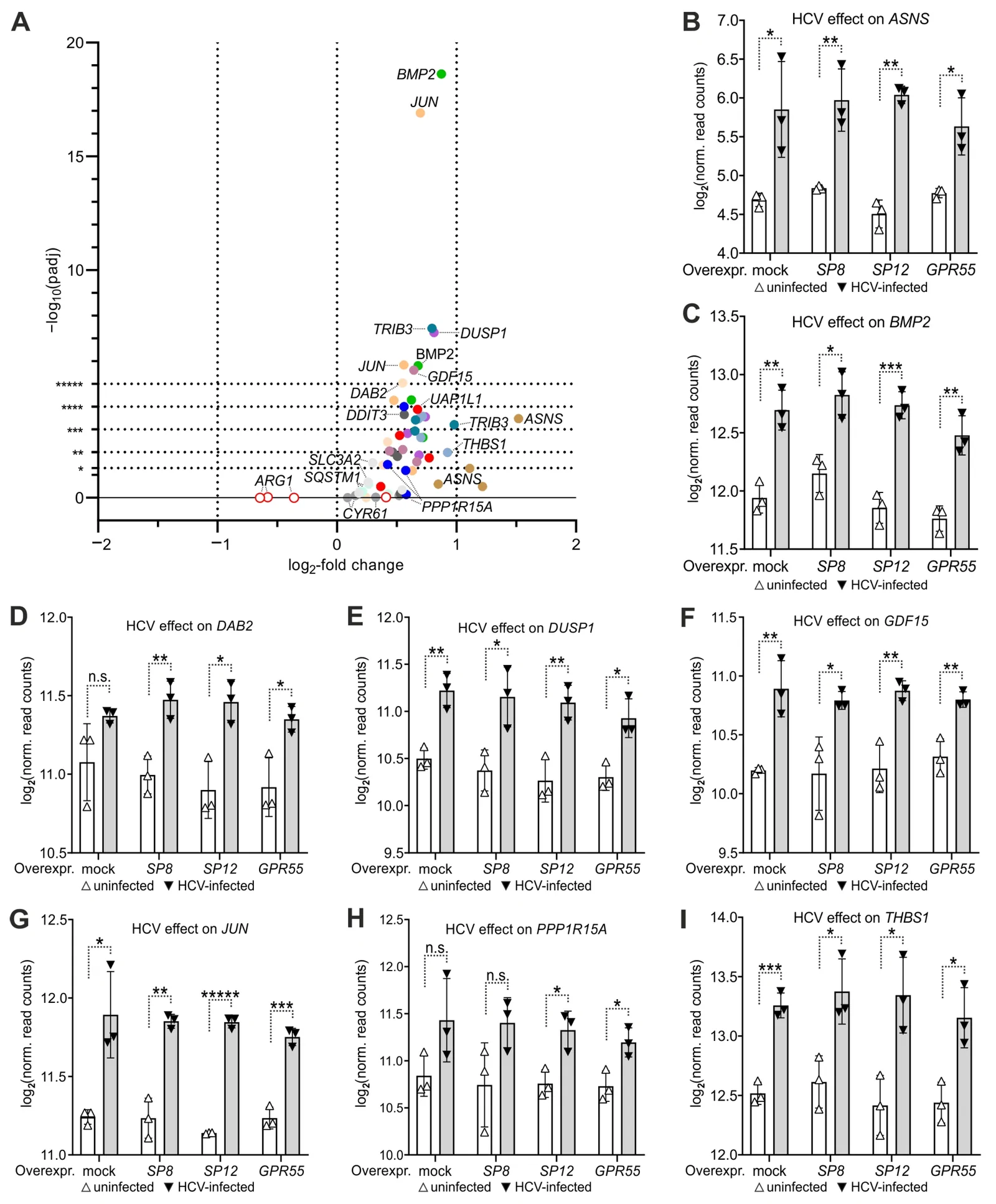
Evaluation of the current NGS data for only those genes that we found differentially regulated by HCV infection in our previous study, except mitochondrial genes. (**A**) Volcano plot of −log_10_(*p*adj) values vs. log_2_-fold changes. Asterisks indicate adjusted *p*-values with * <0.05, ** <0.01, *** <0.001, **** <0.0001 and ***** <0.00001. Each color represents one DEG. The results of *Lnc-ITM2C-1* and *GPR55* overexpression treatments are detailed for selected target genes in (**B**–**I**) (gray bars and black symbols for HCV-infected cells, white bars and symbols for uninfected cells, statistics like in Figure 4B–F).

We then also checked the expression of those genes that we found previously to be upregulated during HCV infection [27]. Even if not all of these genes (Figure 5A) were fulfilling the strict criteria of being significantly induced at least 2-fold in all of the four plasmid overexpression treatment groups, all 14 genes significantly upregulated in our former study are also upregulated here. Induction of eight of these genes under HCV replication is detailed in Figure 5B–I.

*PPP1R15A* (*GADD34*) is part of a phosphatase negatively regulating the stress response [69]. *BMP2*, *DAB2*, *DUSP1*, *JUN* and *GDF15* are involved in growth factor and stress-associated signaling pathways, of which *BMP2* [70] and *GDF15* [71] are ligands of TGF-β superfamily receptors. In addition, *TRIB3* (*SINK*) is an inhibitor of the central transcriptional regulator of innate and adaptive immune responses, NFκB [72]. Again, also in this analysis nearly all genes are moderately or highly expressed (log_2_ values of read counts between 10 and 13.5, compare Figure 3D), except *ASNS*. Notably, also in this set of genes we found no difference in differential gene expression according to the different *Lnc-ITM2C-1* or *GPR55* overexpression conditions.

Taken together, these results are essentially consistent with our former results on cellular genes induced by HCV replication [27].

### 3.4. Lnc-ITM2C-1 and GPR55 Are Not Involved in a Positive Regulatory Circuit During HCV Infection

**Figure 6:**
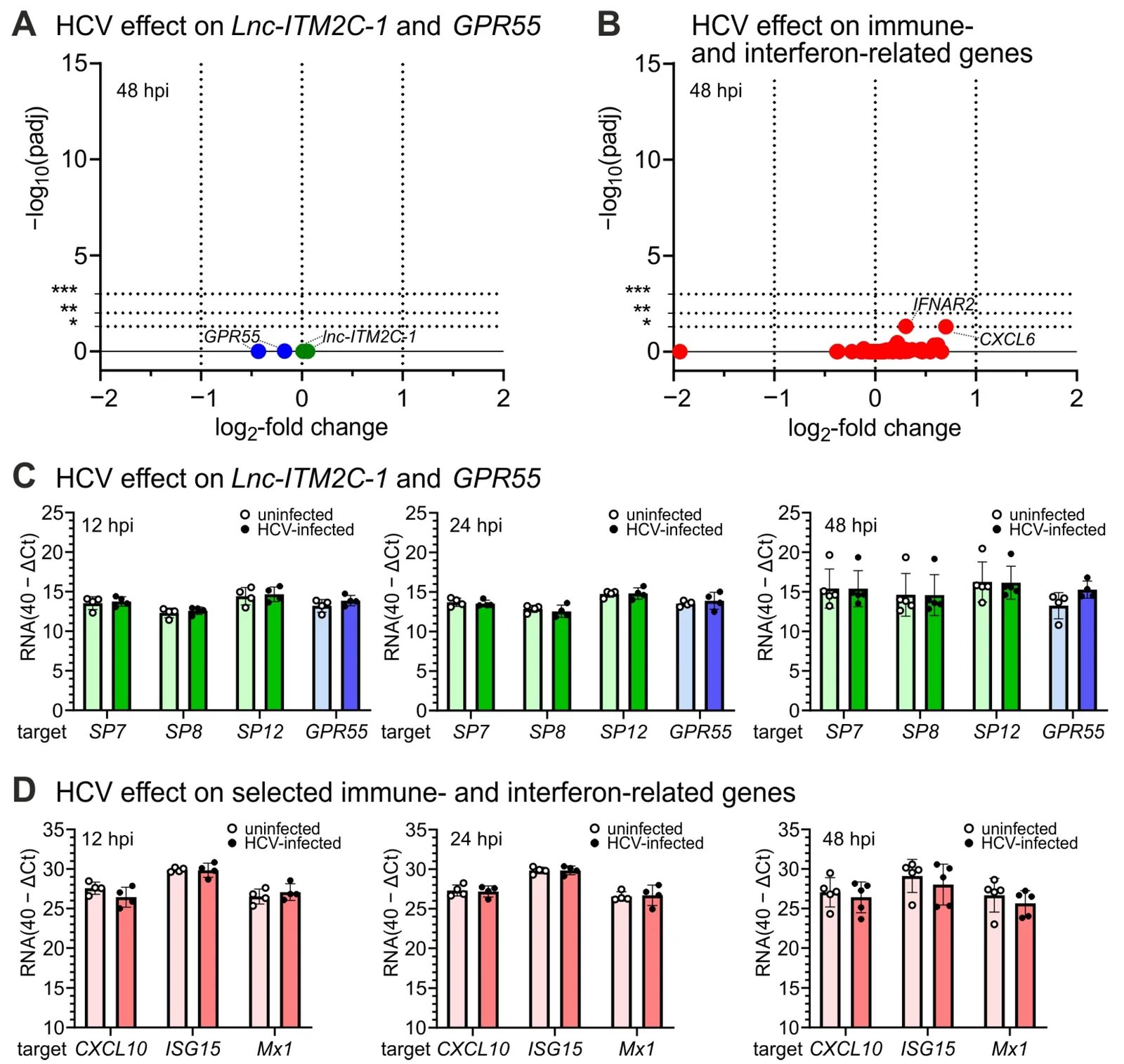
HCV infection did not cause significant change expression of *Lnc-ITM2C-1*, *GPR55* and some selected immune-related genes in Huh-7 Lunet/CD81 cells. (**A**) Influence of HCV infection on endogenous expression of *Lnc-ITM2C-1* (green) and *GPR55* (blue) at 48 hpi. Asterisks indicate adjusted *p*-values with * <0.05, ** <0.01, *** <0.001. (**B**) Influence of HCV infection on those immune- and interferon-related genes (red) that had been tested in our previous study. (**C**) Details of expression of *Lnc-ITM2C-1* splice variants 7, 8 and 12 and *GPR55* mRNA in HCV-infected cells vs. uninfected cells at 12 h post HCV infection (hpi) (left panel), 24 hpi (middle panel) and 48 hpi (right panel). Multiple unpaired *t* tests as well as two-way ANOVA comparisons between uninfected and HCV-infected samples were calculated in GraphPad; all comparisons were not significant. (**D**) Details of the expression of selected ISGs that had been tested in our previous study in HCV-infected and uninfected cells at 12, 24 and 48 hpi. Multiple unpaired *t* tests and two-way ANOVA tests showed that differences between uninfected and HCV-infected cells were not significant.

Based on the consistency of our above results with previous findings, we analyzed whether HCV replication affects expression of *Lnc-ITM2C-1* and *GPR55* RNA, to challenge our previous model of a positive regulation circuit involving HCV and these genes [37]. The results are shown in Figure 6.

To our surprise, we could not find significant stimulation of *Lnc-ITM2C-1* or *GPR55* expression after HCV infection (Figure 6A). Moreover, we also checked the expression of immune- and interferon-related genes (Figure 6B). Only two genes were found to be differentially regulated to a very low extent and with low levels of significance (*CXCL6* and *IFNAR2*). The above results from the NGS experiment were counterchecked by targeted validation using RT-qPCR, with the confirming result that no significant changes in *Lnc-ITM2C-1* and *GPR55* expression were detected according to HCV infection (Figure 6C). In the same way, those immune-related genes that we had reported previously to be downregulated by HCV infection [37] were also not found downregulated in our current experimental setup in Huh-7 Lunet/CD81 cells (Figure 6D).

**Figure 7:**
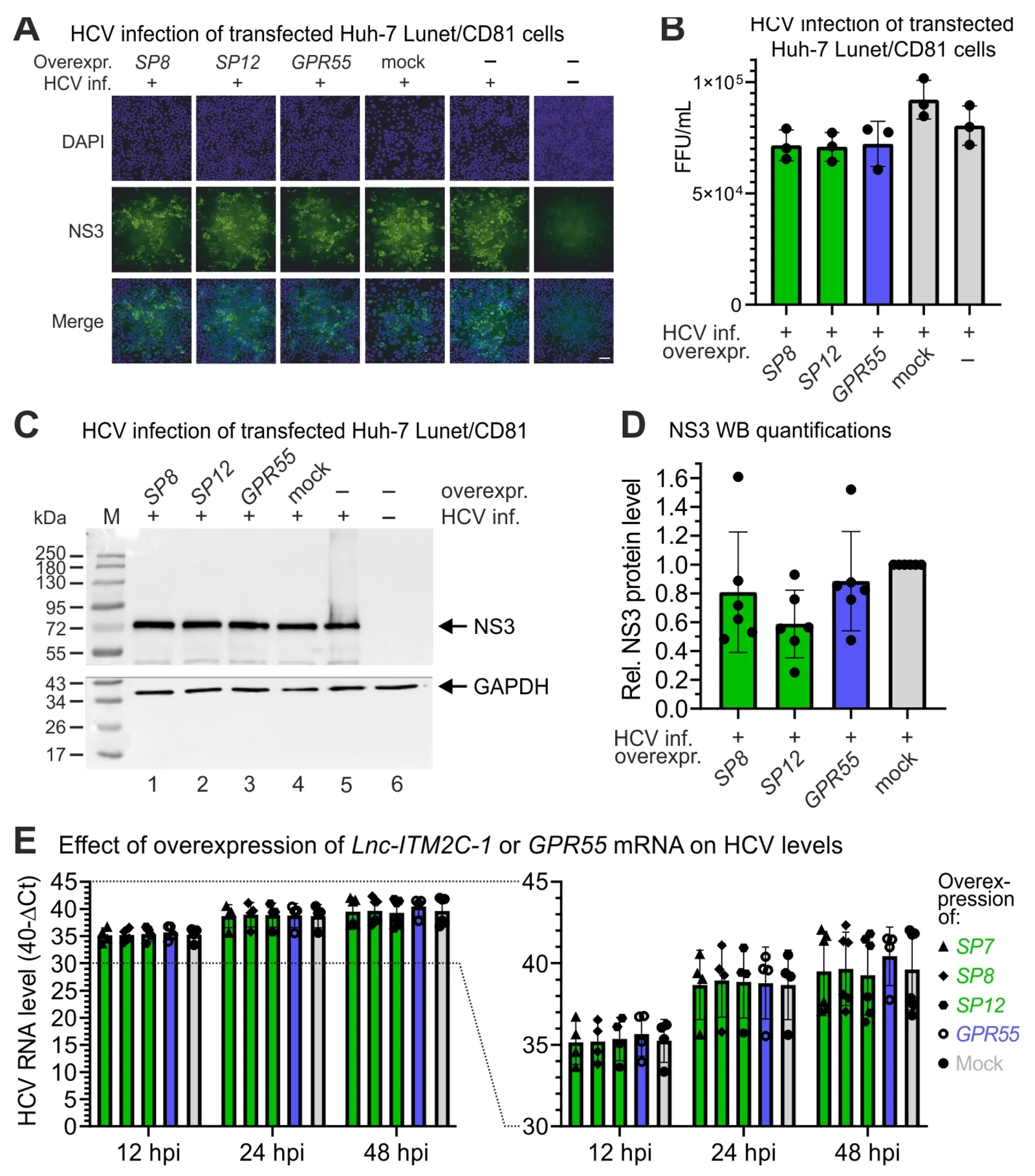
Overexpression of *Lnc-ITM2C-1* or *GPR55* mRNA causes no significant changes in HCV replication and infectious HCV progeny virus production. (**A**) NS3 Immunofluorescence staining of Huh-7-Lunet/CD81 cells at 48 h after infection with supernatant from HCV-infected cells overexpressing *Lnc-ITM2C-1* and *GPR55*, with anti-NS3 antibody (green) and DAPI (blue). The scale bar is 100 µm. (**B**) Focus-forming units (FFU) as a measure for infectious virus particles from the supernatant of HCV-infected Huh-7 Lunet/CD81. Friedman tests followed by Dunn’s multiple-comparison tests were performed for statistical analysis, using mock (empty plasmid) as control. (**C**) Western blot analysis of Huh-7-Lunet/CD81 infected cells, overexpressing *Lnc-ITM2C-1* or *GPR55*, at 48 hpi. GAPDH was used as loading control. (**D**) Quantitative measurement of NS3 protein bands from six independent Western blot experiments. NS3 band intensities were normalized to GAPDH and subsequently normalized to the mock + HCV group. Friedman tests followed by Dunn’s multiple-comparison tests were performed for statistical analysis. (**E**) RT-qPCR analysis of NS3 RNA expression in overexpressing samples at 12, 24, and 48 hpi. Two-way ANOVA tests with Dunnett’s multiple-comparison tests were performed for statistical analysis comparing each overexpression against Mock.

Accordingly, we investigated whether overexpression of these RNAs (*Lnc-ITM2C-1* or *GPR55*) stimulates HCV replication and found no such stimulation (Figure 7A).

Moreover, FFU-based analysis of donor supernatants showed no statistically significant difference in infectious HCV particle production between the overexpression conditions and the control (B). Accordingly, the detection of HCV NS3 protein levels in Western blots (C, D) as well as HCV RNA levels tested by RT-qPCR (E) did not show significant differences in HCV replication under the tested conditions of overexpressing *Lnc-ITM2C-1* and *GPR55* mRNA.

**Figure 8:**
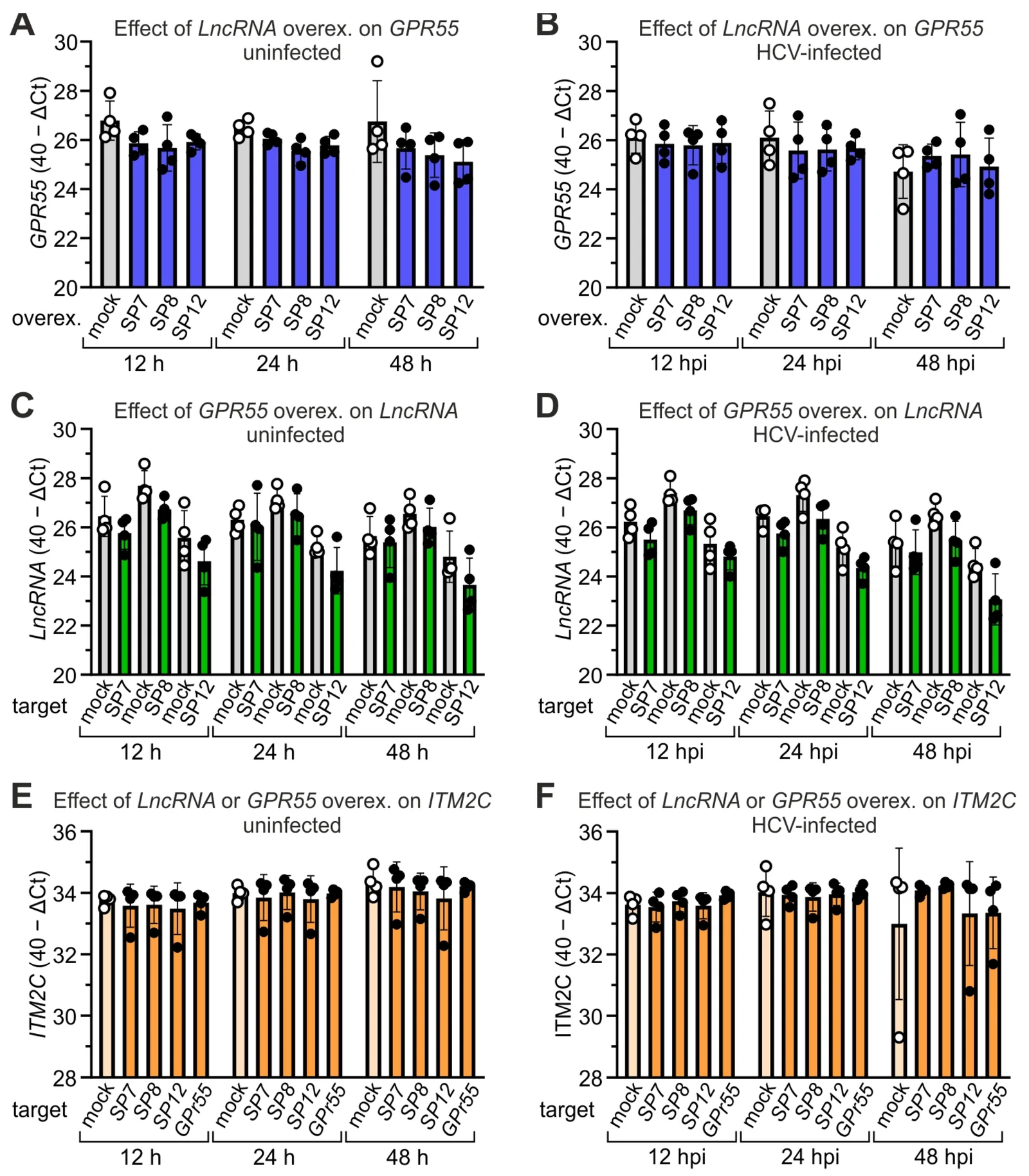
Overexpressed *Lnc-ITM2C-1* or *GPR55* mRNA do not mutually stimulate their gene expression or that of the neighboring ITM2C gene. (**A**,**B**) Analysis of *GPR55* mRNA abundance after overexpression of *Lnc-ITM2C-1* SP7, SP8 and SP12 in uninfected cells (**A**) or 12, 24 or 48 h post HCV infection (hpi) (**B**). (**C**,**D**) Analysis of *Lnc-ITM2C-1* RNA SP7, 8 and 12 abundances after overexpression of *GPR55* in uninfected cells (**C**) or 12, 24 or 48 h after HCV infection (**D**). Analysis of *ITM2C* mRNA abundance after overexpression of *Lnc-ITM2C-1* SP7, 8 and 12 or *GPR55* mRNA in uninfected cells (**E**) or 12, 24 or 48 h after HCV infection (**F**). The data are shown as the means and SD of at least three independent experiments. ANOVA followed by Dunnett’s multiple-comparison test was applied as statistical analysis.

In this context, we also analyzed by RT-qPCR if *Lnc-ITM2C-1* stimulates *GPR55* expression, or if—vice versa—*GPR55* expression stimulates *Lnc-ITM2C-1* expression. Figure 8A shows that overexpression of *Lnc-ITM2C-1* splice variants 7, 8 or 12 did not change *GPR55* mRNA expression, not in uninfected cells (Figure 8A) and not in HCV-infected cells (B). Vice versa, the overexpression of *GPR55* mRNA also did not cause significant changes in the expression of *Lnc-ITM2C-1* splice variant RNAs in uninfected cells (Figure 8C) or HCV-infected cells (D). Moreover, the expression of the neighbor (and name-giving) protein coding gene *ITM2C* (compare Figure 2A) was not affected by *Lnc-ITM2C-1* splice variant expression or *GPR55* mRNA in uninfected (E) or infected cells (F).

Taken together, neither RNA-Seq nor targeted RT–qPCR validation supported the previously proposed positive regulatory circuit involving HCV, *Lnc-ITM2C-1* RNA, and *GPR55* mRNA under the experimental conditions used in this study. These findings suggest that the improved total-RNA purification procedure, including stringent gDNA removal, influenced target detection. Although the mechanism remains unclear, reduced variability attributable to residual gDNA contamination may have contributed.

### 3.5. Analysis of the Effect of Lnc-ITM2C-1 and GPR55 Expression on the Cellular Transcriptome

**Figure 9:**
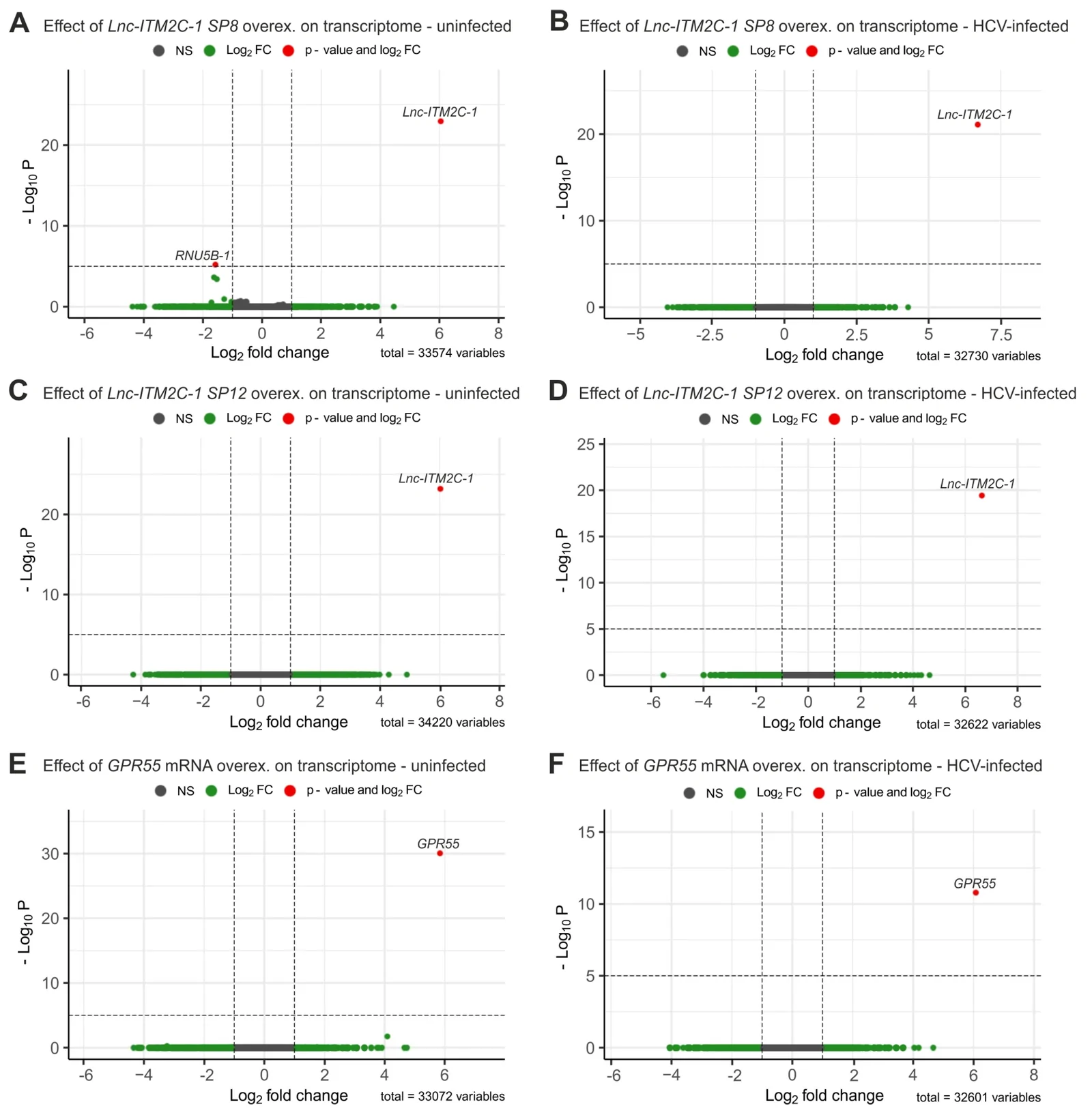
Overexpression of *Lnc-ITM2C-1* or *GPR55* mRNA does not significantly induce cellular genes. (**A**,**B**) Analysis of DEGs after overexpression of *Lnc-ITM2C-1* SP8 in uninfected cells (**A**) or 48 h after HCV infection (**B**). (**C**,**D**) DEGs after overexpression of *Lnc-ITM2C-1* SP12 in uninfected cells (**C**) or 48 h after HCV infection (**D**). DEGs after overexpression of *GPR55* mRNA in uninfected cells (**E**) or 48 h after HCV infection (**F**). Dashed lines indicate LFC = 1 and -log_2_(padj) = 5, respectively.

The overexpression treatment groups were then also analyzed for possible effects of *Lnc-ITM2C-1* SP8, SP12 or *GPR55* mRNA expression on the cellular transcriptome in uninfected cells or HCV-infected cells (Figure 9).

The overexpressed *Lnc-ITM2C-1* or *GPR55* genes are visible about 6 to 7 log_2_ steps overexpressed (i.e., about 64- to 128-fold), consistent with the equivalent results analyzed by RT-qPCR (Supplementary Figure S4F). Besides the gene overexpressed, only one gene in the cellular transcriptome appears significantly differentially regulated under *Lnc-ITM2C-1* SP8 overexpression (see panel A). RNU5B-1, a U5 spliceosomal RNA, was downregulated. Otherwise, no single gene is significantly up- or downregulated under any of the overexpression treatments, not in uninfected and not in HCV-infected cells (Figure 9A–F). Thus, we could not detect any visible effect of the *Lnc-ITM2C-1* or *GPR55* RNA on any gene expressed in the transcriptome of the Huh-7 Lunet/CD81 cells.

### 3.6. Stringent Re-Analysis of Gene Expression Changes During HCV Infection

**Figure 10:**
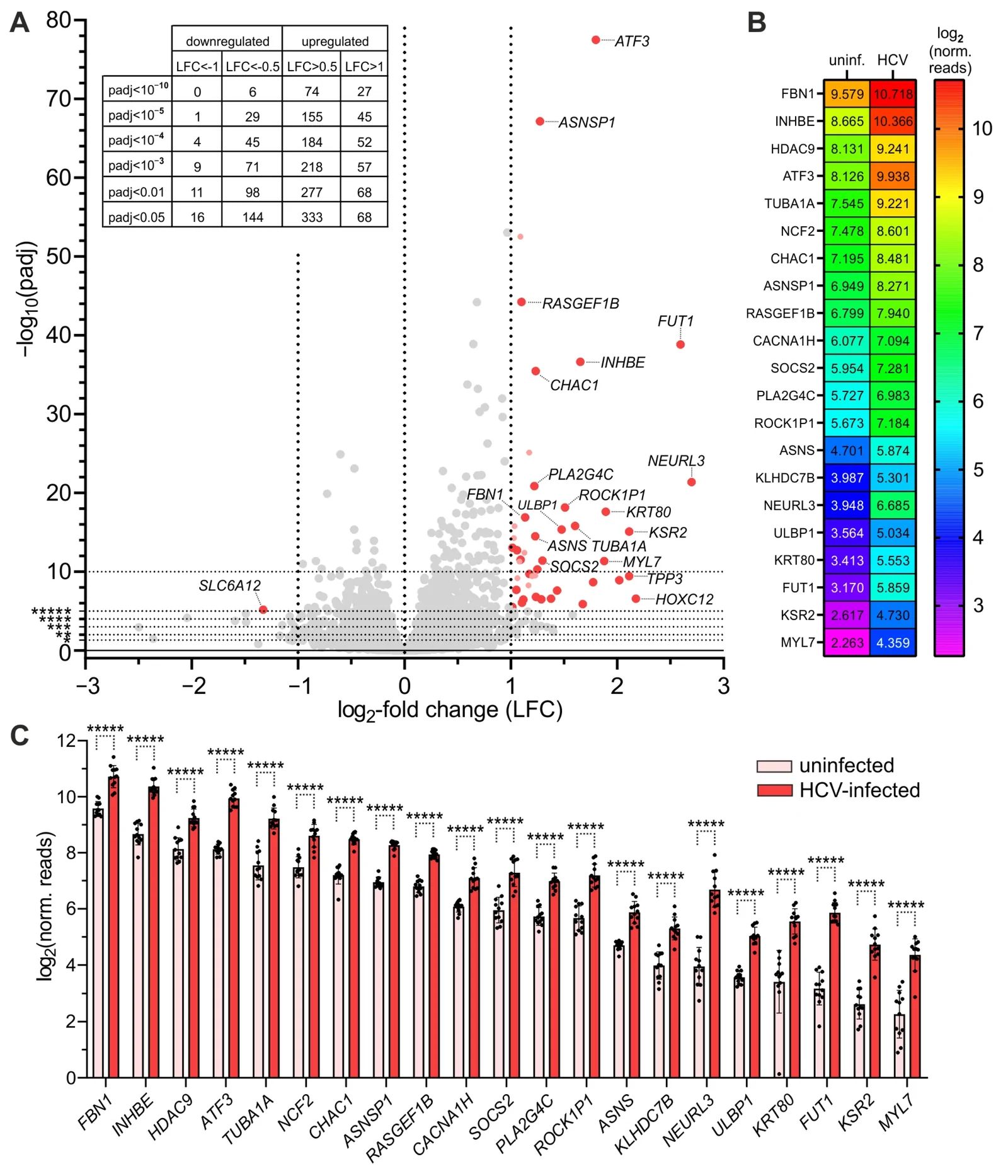
Reevaluation of the RNA-Seq data for the influence of HCV replication on cellular gene expression. Here we combined all 12 replicates from uninfected samples in one group and all 12 replicates from HCV-infected samples in the other group for the analysis of the effect of HCV infection on cellular gene expression. (**A**) Volcano plot as in Figure 4A; here, the values for −log_10_(*p*adj) and log_2_-fold changes (LFC) are combined from 12 HCV-infected samples versus 12 uninfected samples per gene. Large red dots show known genes with −log_10_(*p*adj) > 5 and log_2_-fold changes > 1. Small pink dots show yet uncharacterized genes (“LOC……”). Insert: number of up- and downregulated genes meeting the indicated thresholds. (**B**) Heatmap of the top 21 upregulated characterized genes (not including 6 uncharacterized genes) with *p*adj < 10^−10^ and LFC > 1 (see insert in (**A**)). The values show the means of each 12 uninfected vs. 12 HCV-infected replicates; all replicates are shown in detail in Supplementary Figure S7A. (**C**) Log_2_ (normalized read counts) of those known protein genes and long-non-coding RNA genes from (**A**) with −log_10_(*p*adj) > 10. All 12 replicates are shown with black dots. Significances indicate (not adjusted) *p*-values from *t*-tests of pairwise comparisons with * <0.05, ** <0.01, *** <0.001, **** <0.0001, and ***** <0.00001.

The previous analyses indicated no reproducible significant effects of *Lnc-ITM2C-1* or *GPR55* transcript overexpression on HCV replication or on the cellular transcriptome under the conditions tested. Conversely, *Lnc-ITM2C-1* as well as *GPR55* transcript abundance was not consistently altered by HCV infection. Therefore, we decided to perform a secondary analysis of the above RNA-Seq data (Figure 10) in order to examine the overall transcriptional response to HCV infection in this experimental setting.

To this end, we re-analyzed the above NGS data by combining all 12 RNA-Seq replicate samples from uninfected cells and all 12 replicate samples from HCV-infected cells, irrespective of any of the overexpression treatments (Figure 10). The result is shown in the volcano plot in Figure 10A and for the top 21 characterized genes in the heatmap in Figure 10B. The increased number of samples strongly improved the statistical power and enabled the detection of additional DEGs. In the zone of very strict significance (with two-fold upregulation and an adjusted *p*-value (*p*adj) of < 10^−10^), we now find 21 upregulated characterized genes, instead of only one gene, *ATF3*, in the previous analysis using only 3 replicates each (Figure 4). Consistent with the number of DEGs in the various significance categories (please see top left insert in Figure 10A), this limited effect of HCV infection on cellular gene expression changes is also evident from the corresponding PCA plots (Supplementary Figure S6); when analyzing the top 20, 50 and 100 genes, HCV-infected samples separate clearly from uninfected samples, while overexpression of lncRNA or *GPR55* makes no difference. In contrast, when the top 500 genes are included in the PCA plot calculation, separation becomes less sharp, and the separation disappears with all genes. Genes with a *p*adj < 10^−10^ are detailed in Figure 10C with read counts.

*ATF3* again stands out with an about threefold expression change and outstanding significance. In addition to the genes found above (*CHAC1*, *ASNSP1*, *ROCK1P1* and *TUBA1A*, see Figure 4), we found that inhibin E (INHBE, also called activin E), a ligand of a TGF-β superfamily receptor, is highly significantly induced. The second induced gene involved in lipid metabolism is PLA2G4C, a phospholipase that is involved in lipid droplet formation [73,74]. Three other induced genes are involved in activation of the Ras-MAPK growth factor signaling pathway. RasGEF1B is a guanyl-nucleotide exchange factor that is active in Ras signal transduction [75]. NEURL3 is an inducible E3 ubiquitin ligase with antiviral activity that has been reported to inhibit HCV particle assembly by binding to the viral E1 glycoprotein and disrupting E1–E2 complex formation [76], and it drives MAPK pathway activation [77]. KSR2 is a scaffold protein and weak serine/threonine kinase that organizes RAF-MEK-ERK signaling at the plasma membrane and is involved in activation of the BRAF kinase [78]. Moreover, five genes are involved in microtubule and intermediate-filament cytoskeleton (*TUBA1A*, *MYL7*) or extracellular matrix (*FBN1*, *KRT80* and *FUT1*) organization. Finally, SOCS2 (suppressor of cytokine signaling) is induced. SOCS2 is a subunit of an E3 ubiquitin ligase complex and limits JAK/STAT pathway activity, growth hormone (GH) and insulin-like growth factor (IGF-1) signaling and pro-inflammatory responses [79,80,81]. In addition, 15 more known upregulated genes (Figure 10A, larger red dots) reach a *p*adj of <10^−5^, namely *BHLHE41*, *TPPP3*, *KLHL38*, *ADCYAP1*, *HR*, *CIMAP1C*, *TNFRSF9*, *HOXC12*, *C2CD4C*, *COL1A1*, *MANCR*, *FLRT2-AS1*, *C5orf46* and *ZEB2*, while *SLC6A12* is downregulated.

### 3.7. Changes in Expression of Other lncRNAs During HCV Infection

**Figure 11:**
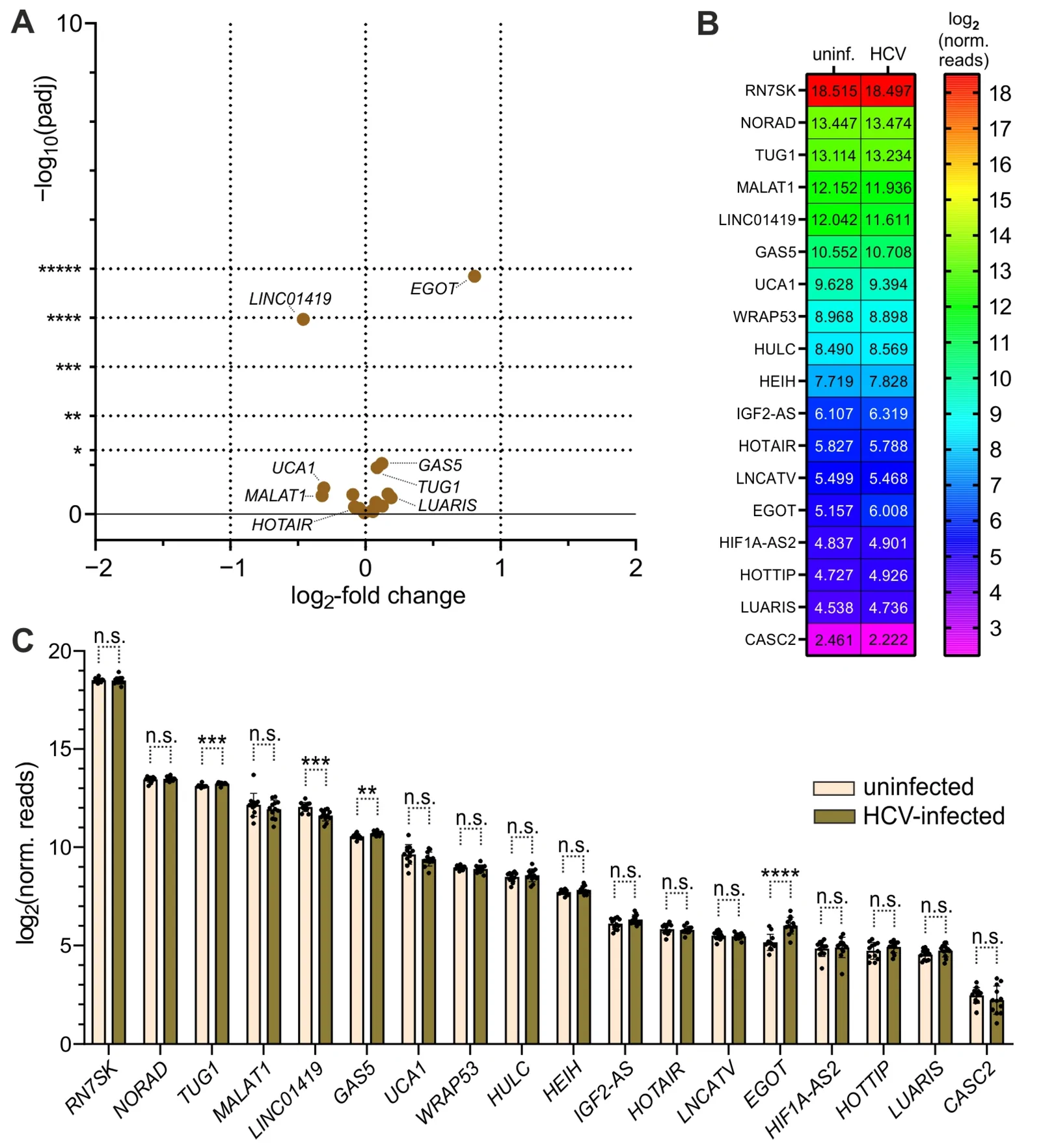
Analysis of the NGS data for expression of 18 selected lncRNAs previously reported to be related to HCV replication. (**A**) Volcano plot as in Figure 10A, showing −log_10_(*p*adj) over log_2_-fold change. (**B**) Heatmap of the above 18 selected lncRNAs (as in Figure 10B). The values show the means of each 12 uninfected vs. 12 HCV-infected replicates; all replicates are shown in detail in Supplementary Figure S7B. (**C**) Individual lncRNA expression, analyzed as in Figure 10B. All 12 replicates are shown with black dots. Significances indicate (not adjusted) *p*-values from *t*-tests of pairwise comparisons with * <0.05, ** <0.01, *** <0.001, **** <0.0001, and ***** <0.00001.

The combined refined dataset of 12 uninfected vs. 12 HCV-infected replicates was then also used to analyze possible changes in expression of those long non-coding RNAs that had been reported to be differentially regulated in the context of HCV replication [82,83,84,85] (Figure 11). The lncRNA EGOT was found to be upregulated in our experiment by about 1.75-fold with high significance, consistent with reports about a positive role of EGOT in HCV replication [82,83,85,86]. lncRNA LINC01419 was mildly but significantly downregulated. Surprisingly, we could not find significant upregulation of several other lncRNAs that had been reported to be upregulated in relation to HCV replication, like HEIC, HOTAIR, HOTTIP, HULC, LUARIS, MALAT1, NORAD and UCA1 [82,83,84,85] in our experimental setting (Figure 11).

## 4. Discussion

In this study, we investigated gene expression changes during Hepatitis C virus infection. HCV replicates slowly [12,13,14,15] and can persist for decades in its host cell [19,20]. Read counts for the HCV genome in infected cells at 48 hpi (i.e., at the peak of fully established replication) constituted only about 2% of the total read counts of a sample, and only about 80 cellular genes were significantly up- or downregulated more than twofold. In this study, we applied stringent RNA purification and gDNA removal procedures in order to allow reliable analysis also of low abundantly expressed genes. Here, we first discuss the DEGs in the context of functional pathways, and then focus on the implications of the stringent RNA purification and gDNA removal for the reliable analysis of low-abundance genes, including lncRNAs.

Among the genes that we found upregulated during HCV infection, in particular four categories of genes that are functionally associated with HCV infection and the HCV-induced cellular response stand out, namely genes involved in 1. ER stress and activation of the UPR, 2. regulation of lipid metabolism, 3. regulation of cytoskeleton organization, and 4. growth regulation and signaling.

In the first category of genes associated with HCV infection is related to ER stress, UPR activation and redox balance [63]. In this context, we find ATF3, CHAC1 and DDIT3/CHOP upregulated. ATF3 is a key transcription factor of the stress response which can limit NFκB activity [64,66,87,88], DDIT3/CHOP is involved in restoring the normal function of protein folding capacity [67,68], and CHAC1 degradates intracellular glutathione in order to maintain the intracellular glutathione redox balance [67,68]. Also TRIB3, PPP1R15A [27,89] and INHBE [90] help to limit ER stress. Thus, the above genes might be involved in moderating ER stress caused by HCV replication. The second category includes genes that control lipid metabolism. The phospholipase PLA2G4C supports HCV replication and assembly by membrane phospholipid remodeling and lipid droplet formation [73,74,91,92,93,94], whereas Inhibin E (activin E) and GDF15 appear to be involved in limiting lipid mobilization [95,96,97]. The third category is intertwined with the above regulation of ER stress, UPR, lipid metabolism and lipid droplet formation involved in HCV assembly [92,93,94] in terms of upregulation of cytoskeleton-associated components, including tubulin 1A (TUBA1A), myosin light chain 7 (MYL7) and keratin 80 (KRT80). Also RASGEF1B may contribute to HCV assembly [75] by regulating cytoskeletal rearrangements [92,93,94,98]. The fourth category includes genes that may contribute to growth regulation and signaling, like the transcription factor JUN [99,100] and KSR2 that can activate the BRAF kinase in the MAPK pathway [78]. In contrast, the ubiquitin ligase NEURL3 (LINCR) may limit HCV replication [76] and promote inflammatory responses through degradation of MAPK-phosphatase 1 (MKP1) [77]. Also SOCS2 can limit cytokine receptor signaling through the JAK-STAT pathway [64,101].

Taken together, such inhibition of HCV replication and moderation of the perturbation of cellular lipid metabolism and the cellular innate responses may contribute to the establishment of HCV’s low level replication and persistence. In other words, this balanced response could allow HCV to remain replicating in the cell without, on the one hand, causing severe cell death or, on the other hand, being subject to complete virus elimination.

Our above results on the regulatory pathways affected by HCV infection are largely consistent with previous studies [31,32,33,34,35]. However, it is important to note that several genes that were found significantly upregulated (*SOCS2*, *PLA2G4C*, *ASNS*, *NEURL3* and *KSR2*) are low abundantly expressed genes, with log_2_ values of read counts between 2 and 7, i.e., in the lower body of the violins in Figure 3D, while they might be involved in the regulation of important signaling cascades. All these genes have counts of “Reads Per Kilobase of transcript per Million of mapped reads” (RPKM) between 0.01 and 0.75 and thus would have been neglected by studies limiting their analyses to genes with RPKM values > 1. Therefore, the reliable detection of significant changes also of low abundantly expressed genes may be useful for the complete understanding of gene expression changes involved in response to infection with a slowly replicating virus like HCV.

Looking at the technical implications of our study, purification of total RNA from cells using three additional steps for gDNA removal after TRIzol extraction resulted in a decrease in gDNA contamination by at least 9 or 10 log_2_ steps (please see the noRT controls in Figure 1), i.e., corresponding to a factor of approximately 500 to 1000. This, in combination with the use of random RT primers, appropriate RT annealing temperature protocols and well-tested (q)PCR primers (≥90% amplification efficiency, single sharp melting-curve peak, and one gel band of the expected size), allowed the specific and reliable detection of splice variant RNAs of *Lnc-ITM2C-1* and *GPR55* mRNA.

Previous gene expression analyses showed increasing low-level fluctuations with increasing gDNA contamination [39]. Such gDNA contamination was present in those RNA preparation samples that had undergone either no DNase treatment or other gDNA removal step [39]. Such results may have contributed to the widespread assumption that gDNA contamination causes an inflation of low-level reads. In contrast, Liu and coworkers [40] showed that the gene expression read count output from poly(A)^+^-RNA samples that are devoid of gDNA has a bimodal distribution. In RNA-Seq samples with detectable contaminations by intergenic region and intron sequences, which are indicators of gDNA contamination (Figure 2A and Figure S6A in [40]), the box plots showed an upward shift in the median of gene expression values (Figure 2B and Figure S6B in [40]), coincident with the lack of a bimodal read count distribution and the (relative) loss of lower read counts (Figure 2C and Figure S6C in [40]). In contrast, samples in [40] with low gDNA contamination (i.e., low proportions of reads mapping to intergenic regions and introns) showed a bimodal-like read count distribution including a remarkable density of low expression genes.

Consistent with the above findings of Liu and coworkers [40], our samples (violin plots in Figure 3D) show a bimodal read count distribution, with an overall median at a log_2_ value of about 7 (i.e., about 128 read counts for a given gene), a broad upper part centered at about 10, but also a broad lower part centered at about 4. In both sections, there are genes that are expressed at very similar levels in all replicates, irrespective of overexpression treatment or HCV infection. In the upper section these include several known reference genes (Figure 3F). In the lower section, we did not find recommended reference genes, but we found low-abundance protein coding RNAs (right part of Figure 10C) and non-coding RNAs (right part of Figure 11C) expressed highly reproducibly among all replicates. *Lnc-ITM2C-1* is endogenously expressed at even lower levels, with 2 read counts (log_2_ (read counts) = 1) on average (Figure 3I). Thus, the bimodal-like distribution observed in our samples may coincide with low apparent gDNA contamination and preservation of a reproducible low-expression gene population. In contrast, the violin plots of the log_2_ (read counts) of another previous NGS experiment from 2021 (shown in Figure 3E, not further used in this study), in which we did not use improved gDNA removal, show a read count distribution with fewer reads from low abundantly expressed genes, consistent with those samples that contained a high gDNA content in [40].

We hypothesized that the better removal of gDNA from our samples might lead to a higher proportion of exonic vs. non-exonic reads (the latter largely representing intronic reads) in annotated genes. To our surprise, re-running featureCounts in order to count exonic vs. non-exonic reads from both different RNA-Seq experiments showed a higher (apparent) fraction of exonic reads of 81.8% in the previous 2021 experiment (Supplementary Table S5A, red) compared with our current study (71.2%, blue). However, the parameters for data evaluation were different between the two RNA-Seq experiments, regarding the human genome version, gene annotation, as well as other parameters and filtering steps.

Therefore, we analyzed from both RNA-Seq datasets the number of those biological replicates in which at least some reads were found for the lncRNAs listed in Figure 11B,C (# reads > 0) (Supplementary Table S5B). In our current dataset, we found that in all replicate samples reads for all lncRNAs listed in Figure 11 were found. Regarding *Lnc-ITM2C-1*, the read count was 0 in only one replicate. In contrast, in the previous RNA-Seq dataset from 2021 we found that for highly expressed lncRNAs (see left part of Figure 11C), reads were detected in all biological replicate samples, but for the low-abundance expressed lncRNAs we found that in several or even all replicates the respective lncRNA was not detected at all (in particular *LNCATV*, *HOTTIP* and *LUARIS*) (Supplementary Table S5B, lower right part in red). Also *Lnc-ITM2C-1* was actually not contained at all in the gene lists from the previous 2021 experiment. We can only speculate that, besides the fact that the two data evaluation pipelines may have been slightly different, insufficient gDNA removal may lead to differences in filtering of low-level reads by the evaluation pipeline [39,54].

By using our improved RNA purification protocol, *Lnc-ITM2C-1* and *GPR55* mRNA revealed not to be involved in a positive regulation circuit during HCV infection as we had reported previously [37]. A possible reason may be that in that previous study, we had used standard TRIzol RNA preparation, directly followed by DNase I treatment, then followed by a GeneJET RNA Clean-up Kit. However, since the aqueous supernatant of phenol–water mixes contains about 7% phenol [46], traces of phenol might be carried over to the pellet formed after precipitation of the RNA. This phenol may disable the action of enzymes like DNase I when applied as the very next step. Thus, in our previous study varying amounts of gDNA contamination may have inappropriately influenced the results. In contrast, now we used a silica-matrix column purification step before DNase treatment (removing residual phenol before using DNase), and we have used three such purification steps after the TRIzol extraction step (Figure 1).

In this low expression range we also found some well-known lncRNAs very consistently detected in all biological samples, like *IGF2-AS*, *HOTAIR*, *LNCATV*, *HOTTIP* and *LUARIS* (Figure 11C). This suggests that these low expression values reflect genuine RNA expression similar in all 24 biological replicates rather than fluctuating technical artifacts due to varying inflation of samples by gDNA contamination [38].

A limitation of our study may be considered that we used an MOI of 0.3. This may have reduced the effect strength in terms of expression fold change in comparison with the use of higher MOIs in which a higher fraction of cells undergoes the HCV-induced gene expression changes at the same time. Nevertheless, we find 477 DEGs using mild thresholds for fold change and significance like they were also used in other studies [31,32]. Interestingly, some of these studies also used MOIs of 0.02, 0.05 or 0.5 [31,32,34], and the results yielded a comparable number of DEGs that are involved in similar pathways. Thus, a possible coexistence of freshly established replicating HCV genomes and repeated round-replication of HCV genomes in the cells does not appear to have a strong disturbing impact on the largely consistent detection of targeted DEGs as a response to the HCV infection.

In conclusion, for the investigation of low-level expressed genes by RNA-Seq or RT-qPCR, we strongly recommend stringent gDNA removal (like by our improved RNA purification protocol), in combination with stringent validation (see Figure 1; noRT control, and gels for checking correct product size). This may be of particular importance for the investigation of low-abundance expressed long non-coding RNAs and other genes.

## Data Availability

Data are contained within the article and Supplementary Materials.

## Supplementary Materials

The following supporting information can be downloaded at: https://www.mdpi.com/article/10.3390/v18101167/s1, Khajouei_RNA-seq sample name decoding table.pdf. Khajouei_RNA-seq_Gene_counts_and_log2_normalized.xlsx. Khajouei_RNA-seq_Lists of HCV-induced DEGs-related to Figure 10.xlsx. Khajouei_RNA-seq_RPKM_reads_per_kilobase_of_transcript_per_million_mapped_reads.xlsx. Khajouei_Splice variants and successful primers-GPR55.dna. Khajouei_Splice variants and successful primers-Lnc-ITM2C-1.dna. Khajouei_Supplementary Figures S1–S7-Supplementary Tables S1–S5.docx. Khajouei_Supplement_multiqc_report.html. Khajouei_Transcripts and Chromosome Nt numbers-Lnc-ITM2C-1 and GPR55.docx.

## Abbreviations

The following abbreviations are used in this manuscript (for gene names, please also refer to “The Human Gene Database”; “GeneCards”; https://www.genecards.org/, accessed on 10 September 2026):

3′UTR	3′Untranslated Region
5′UTR	5′Untranslated Region
CD81	Cluster of Differentiation 81, a cell surface protein
Ct	Cycle threshold
ΔCt	Difference of cycle thresholds, Ct (gene of interest) −Ct (reference gene)
EMCV	Encephalomyocarditis Virus
FFU	Focus Forming Units (equivalent to infectious virus particles)
gDNA	genomic DNA
GPR55	G-Protein Coupled Recepter 55
HCV	Hepatitis C Virus
hpi	hours post-infection
hpt	hours post-transfection
Huh-7	human hepatoma cell line
IRES	Internal Ribosome Entry Site
ISG(s)	Interferon-stimulated gene(s)
ITM2C	Integral Membrane Protein 2C
Lnc-ITM2C-1	long non-coding RNA ITM2C-1
lncRNA	long non-coding RNA (>200 b)
log_2_FC	log_2_-fold change
MANE	Matched Annotation from NCBI and EMBL-EBI
NS3	Non-Structural Protein 3 of HCV
overex(pr).	overexpression
*p*adj	adjusted *p*-value
qPCR	quantitative (Real-Time) Polymerase Chain Reaction
RefSeq	Reference SequenceReverse Transcriptase or Reverse Transcription
RT	Reverse Transcriptase or Reverse Transcription
SP	Splice variant
