# Supplementary material for: Transcriptome Analysis of Hepatitis C Virus-Infected Hepatoma Cells Following Stringent Removal of Genomic DNA
Source: Viruses. 2026 Oct 20;18(10):1167. doi: 10.3390/v18101167 (PMC13709437; doi:10.3390/v18101167)
Supplement: Supplementary file 1 [file viruses-18-01167-s001.zip › Khajouei_RNA-seq sample name decoding table.pdf]

Khajouei et al. RNA-seq sample name decoding table

Treatment groups = overexpression plasmid	Biological replicate	HCV infection	Naming in text and figures	Naming in DESeq2
empty plasmid = mock	1	no	mock_R1	IRES_EGFP_B1
empty plasmid = mock	2	no	mock_R2	IRES_EGFP_B2
empty plasmid = mock	3	no	mock_R3	IRES_EGFP_B3
lnc-ITM2C-1 splice var. 8	1	no	SP8_R1	sp8_B1
lnc-ITM2C-1 splice var. 8	2	no	SP8_R2	sp8_B2
lnc-ITM2C-1 splice var. 8	3	no	SP8_R3	sp8_B3
lnc-ITM2C-1 splice var. 12	1	no	SP12_R1	sp12_B1
lnc-ITM2C-1 splice var. 12	2	no	SP12_R2	sp12_B2
lnc-ITM2C-1 splice var. 12	3	no	SP12_R3	sp12_B3
GPR55 mRNA	1	no	GPR55_R1	GPR55_B1
GPR55 mRNA	2	no	GPR55_R2	GPR55_B2
GPR55 mRNA	3	no	GPR55_R3	GPR55_B3
(empty plasmid)	1	yes	HCV_mock_R1	IRES_EGFPplusHCV_B1
(empty plasmid)	2	yes	HCV_mock_R2	IRES_EGFPplusHCV_B2
(empty plasmid)	3	yes	HCV_mock_R3	IRES_EGFPplusHCV_B3
lnc-ITM2C-1 splice var. 8	1	yes	HCV_SP8_R1	sp8plusHCV_B1
lnc-ITM2C-1 splice var. 8	2	yes	HCV_SP8_R2	sp8plusHCV_B2
lnc-ITM2C-1 splice var. 8	3	yes	HCV_SP8_R3	sp8plusHCV_B3
lnc-ITM2C-1 splice var. 12	1	yes	HCV_SP12_R1	sp12plusHCV_B1
lnc-ITM2C-1 splice var. 12	2	yes	HCV_SP12_R2	sp12plusHCV_B2
lnc-ITM2C-1 splice var. 12	3	yes	HCV_SP12_R3	sp12plusHCV_B3
GPR55 mRNA	1	yes	HCV_GPR55_R1	GPR55plusHCV_B1
GPR55 mRNA	2	yes	HCV_GPR55_R2	GPR55plusHCV_B2
GPR55 mRNA	3	yes	HCV_GPR55_R3	GPR55plusHCV_B3
