# Supplementary material for: Transcriptome Analysis of Hepatitis C Virus-Infected Hepatoma Cells Following Stringent Removal of Genomic DNA
Source: Viruses. 2026 Oct 20;18(10):1167. doi: 10.3390/v18101167 (PMC13709437; doi:10.3390/v18101167)
Supplement: Supplementary file 1 [file viruses-18-01167-s001.zip › Khajouei_Supplementary Figures S1–S7 - Supplementary Tables S1–S5.pdf]

Transcriptome analysis of Hepatitis C Virus-infected hepatoma cells following stringent removal of genomic DNA, Mozhdeh Khajouei et al.

Supplementary Figure S1-related to Figure 1B–D

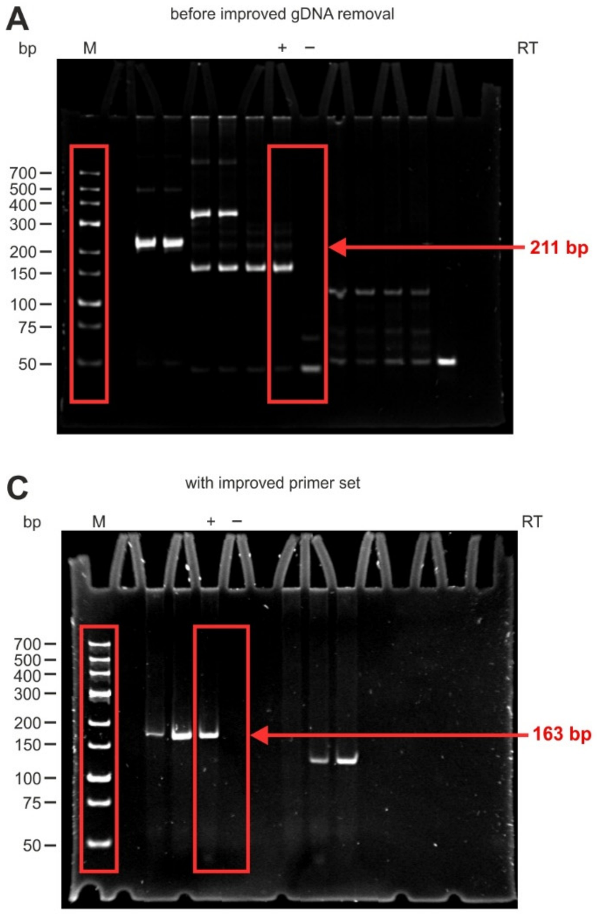

D

RT and noRT Ct values for Fig. 1B, C, D and Suppl. Fig. 1E						
**Figure**	**RNA purification method**	**Target/primer set**	**RT Ct**	**noRT Ct**	**ΔCt (noRT-RT)**	**Comment**
Fig.1B	Standard TRIzol extraction	Lnc-ITM2C-1 all variants except 4/5/6/8/14/15 exon 1 common/PS1$_a$	22.84	34.43	11.59	gDNA contamination, use of gene-specific primers for cDNA synthesis
Fig.1C	TRIzol + acidic phenol + NEB column + 1x rDNase	Lnc-ITM2C-1 all variants except 4/5/6/8/14/15 exon 1 common/PS1	21.36	> 40	> 18.64	Improved gDNA removal, but high Ct for the low-expressed gene indicated limited assay specificity, prompting primer optimization
Fig.1D	TRIzol + acidic phenol + NEB column + 1x rDNase	Lnc-ITM2C-1-SP7/PS3	34.33	> 40	> 5.67	No gDNA contamination, use of random primers for cDNA synthesis
Suppl. Fig. 1E	TRIzol + acidic phenol + NEB column + 1x rDNase	GAPDH for + GAPDH rev	24.50	> 40	> 15.40	No gDNA contamination, random primers for cDNA
Suppl. Fig. 1E	TRIzol + NEB column + 2x rDNase	GAPDH for + GAPDH rev	24.42	> 40	> 15.38	No gDNA contamination, random primers for cDNA

Supplementary Figure S1 - continued - related to Figure 1B–D

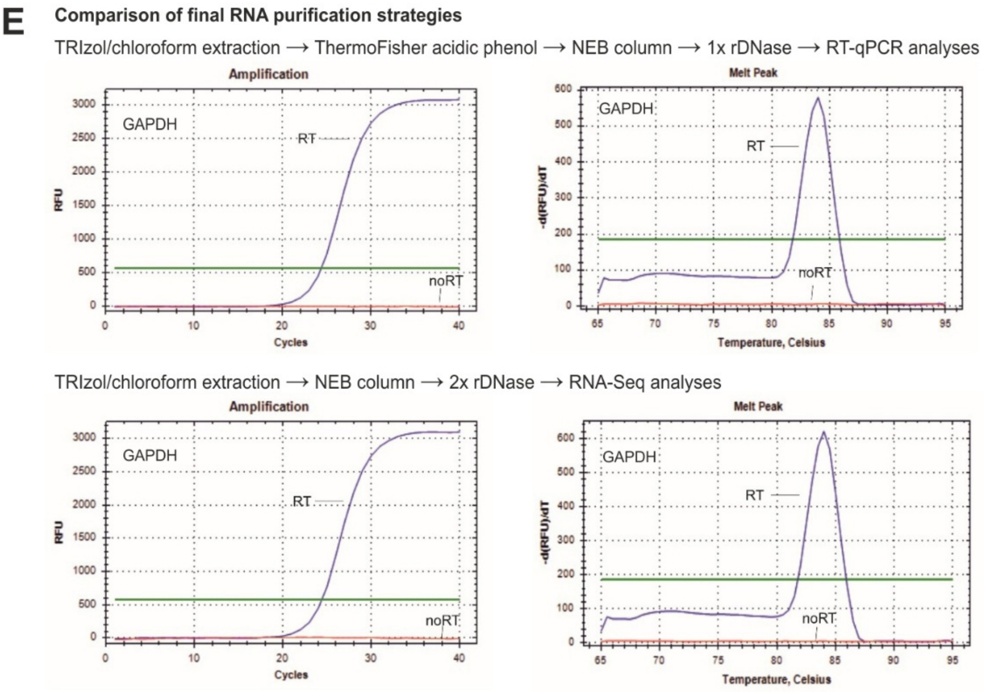

Supplementary Figure S2-related to Figure 2C

A related to Fig. 2C, left panel - *Lnc-ITM2C-1* - PS3a

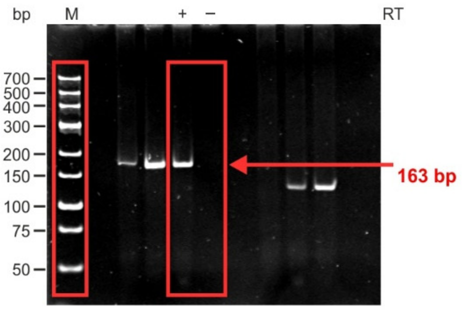

B related to Fig. 2C, middle panel - *Lnc-ITM2C-1* - PS9

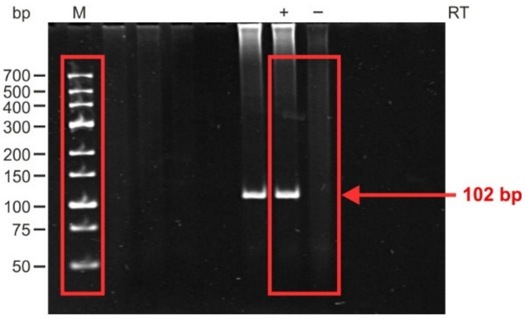

C related to Fig. 2C, right panel - *Lnc-ITM2C-1* - PS10f

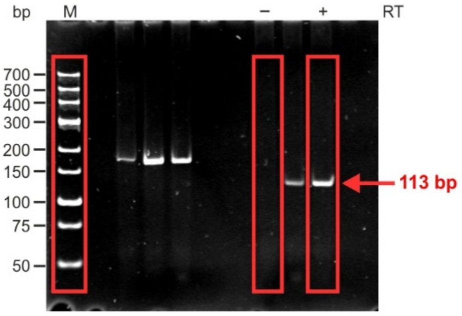

D **Representative RT and noRT Ct values for Lnc-ITM2C-1/GPR55 Fig 2**

**RT and noRT Ct values for Fig. 2. C-E**					
**primer set**	**RNA purification method**	**Target**	**RT Ct**	**noRT Ct**	**ΔCt (noRT-RT)**
**Lnc-ITM2C-1 primer sets are represented in Fig.2**					
PS3a	Acidic phenol + NEB column + 1x rDNase	Lnc-ITM2C-1-SP7	34.33	> 40	> 5.67
PS9		Lnc-ITM2C-1 variants 2/4/5/7/8-exon 2 common	34.41	> 40	> 5.59
PS10f		Lnc-ITM2C-1-SP12	35.47	> 40	> 4.53
**GPR55 primer sets are represented in Fig.2**					
PS4	Acidic phenol +NEB column + 1x rDNase	GPR55-SP3/11	35.68	> 40	> 4.32
PS5		GPR55-all splice variants	33.63	> 40	> 6.37
PS10		Indirectly GPR55-SP1 (SP1/6/7/8/9 directly)	34.32	39.95	5.63

Supplementary Figure S3-related to Figure 2E

A related to Fig. 2E, left panel - *GPR55* - PS4

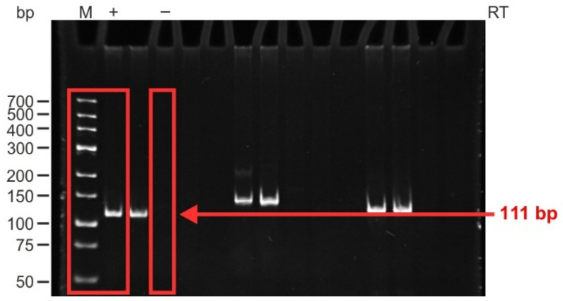

B related to Fig. 2E, middle panel - *GPR55* - PS5

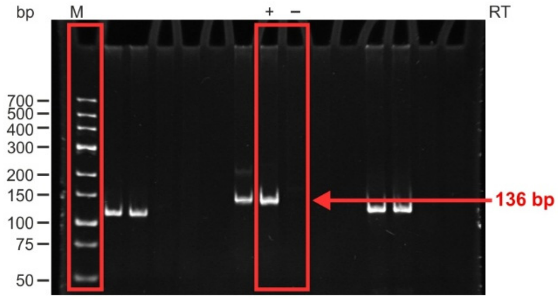

C related to Fig. 2E, right panel - *GPR55* - PS10

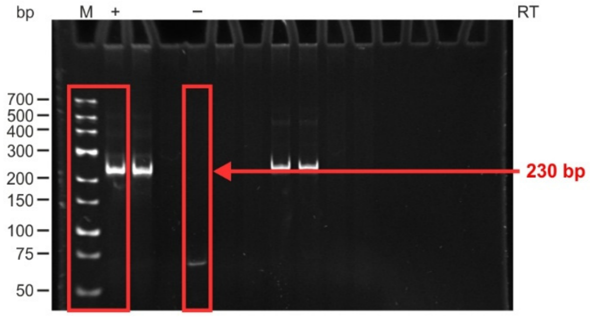

D related to Fig. 2E, right panel - *GPR55* - PS10 - additional RT-qPCR experiment

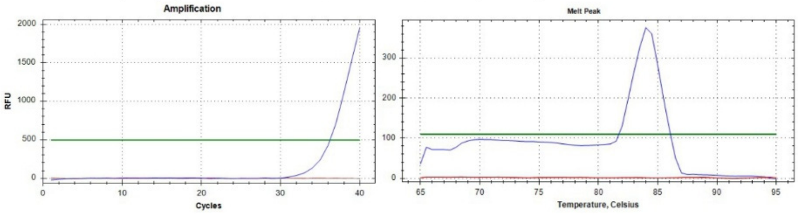

Supplementary Figure S4-related to Figure 3 – pre-RNA-Seq tests

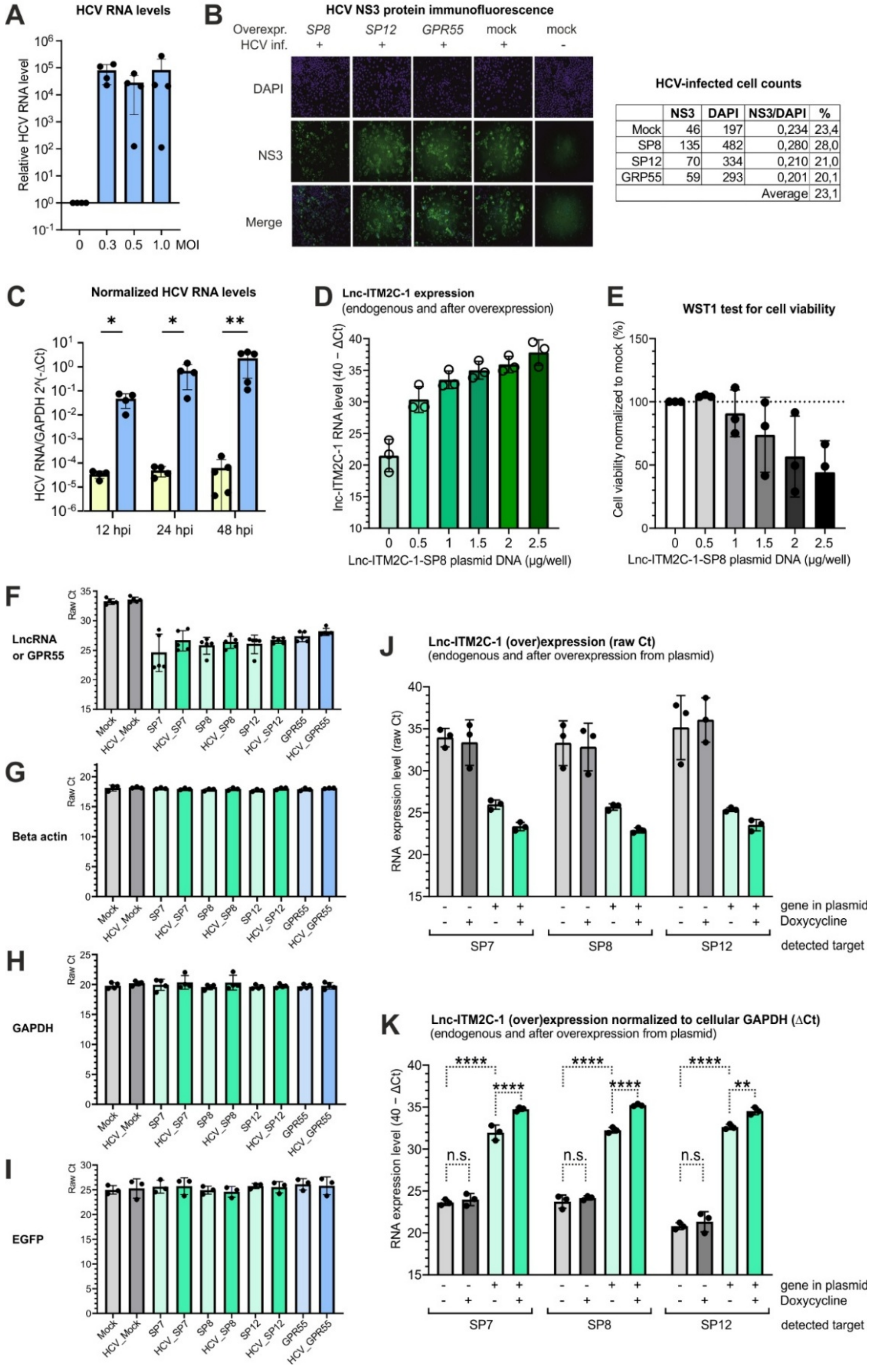

	NS3	DAPI	NS3/DAPI	%
Mock	46	197	0,234	23,4
SP8	135	482	0,280	28,0
SP12	70	334	0,210	21,0
GRP55	59	293	0,201	20,1
			Average	23,1

(A) HCV RNA levels measured by RT-qPCR at different multiplicities of infection (MOIs). **(B)** Immunofluorescence and counts of cells stained with an NS3 antibody and with DAPI stain, the NS3/DAPI ratio is the fraction of infected cells. **(C)** Time course of HCV infection; Ct values of HCV RNA levels were normalized to GAPDH mRNA Ct values. Means and standard deviations (SDs) are shown, significances are p-values from T-tests of pairwise comparisons with * $p < 0.05$, ** $p < 0.01$. For the following RNA-seq experiment, RNA was isolated from the cells at 48 h post infection (hpi). **(D)** Lnc-ITM2C-1 espression levels normalized to GAPDH after transfection of different amounts of plasmid DNA to 6-well plates. **(E)** WST-1 cell viability related to (C). **(F-I)** Raw Ct values of Lnc-ITM2C-1 splice variants and GRP55 mRNA (F), in comparison to reference genes β-Actin (G), GAPDH (H) and the internal plasmid reference gene EFGP (I). **(J)** Raw Ct values of expression of endogenous Lnc-ITM2C-1 splice variants 7, 8 and 12 (light grey bars) and the RNAs overexpressed from the respective gene in the plasmid (green bars), either in the absence of doxycycline (light colour) or in the presence (dark colour). **(K)** Data from (J) normalized to GAPDH (ΔCt) and expressed as 40-ΔCt values.

Supplementary Figure S5-related to Figure 3

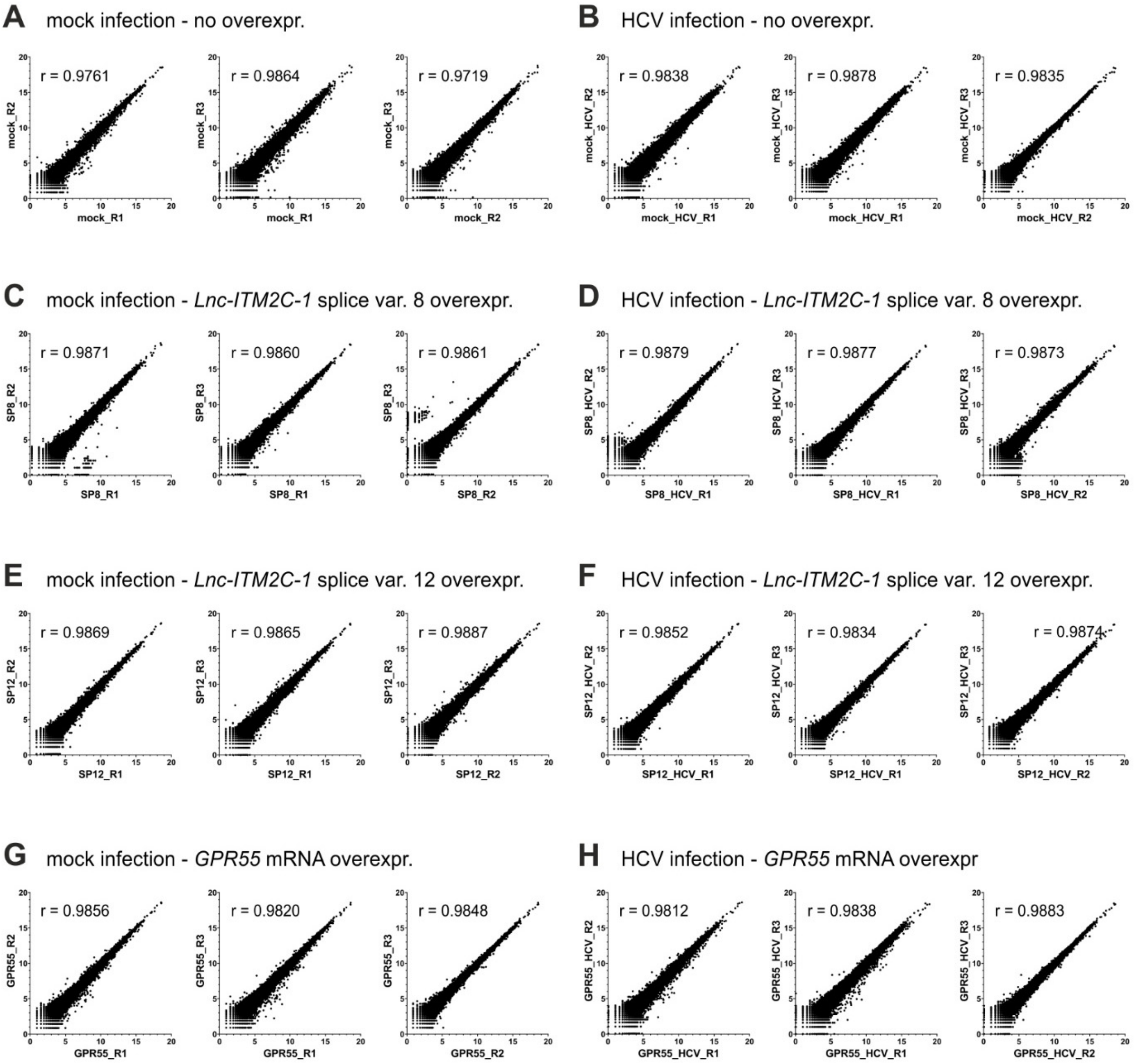

Supplementary Figure S6-related to Figure 10A

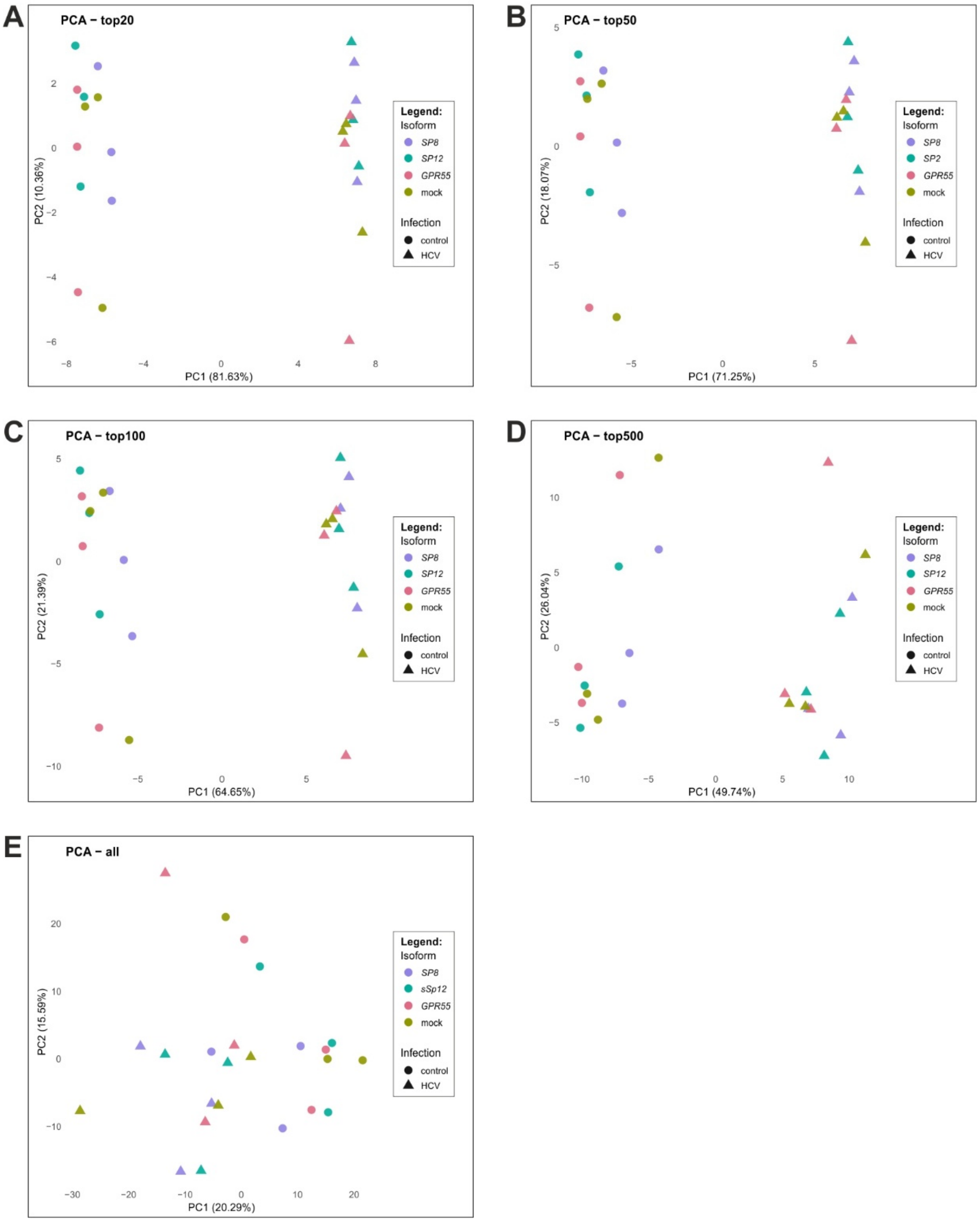

Supplementary Figure S7A-related to Figure 10B

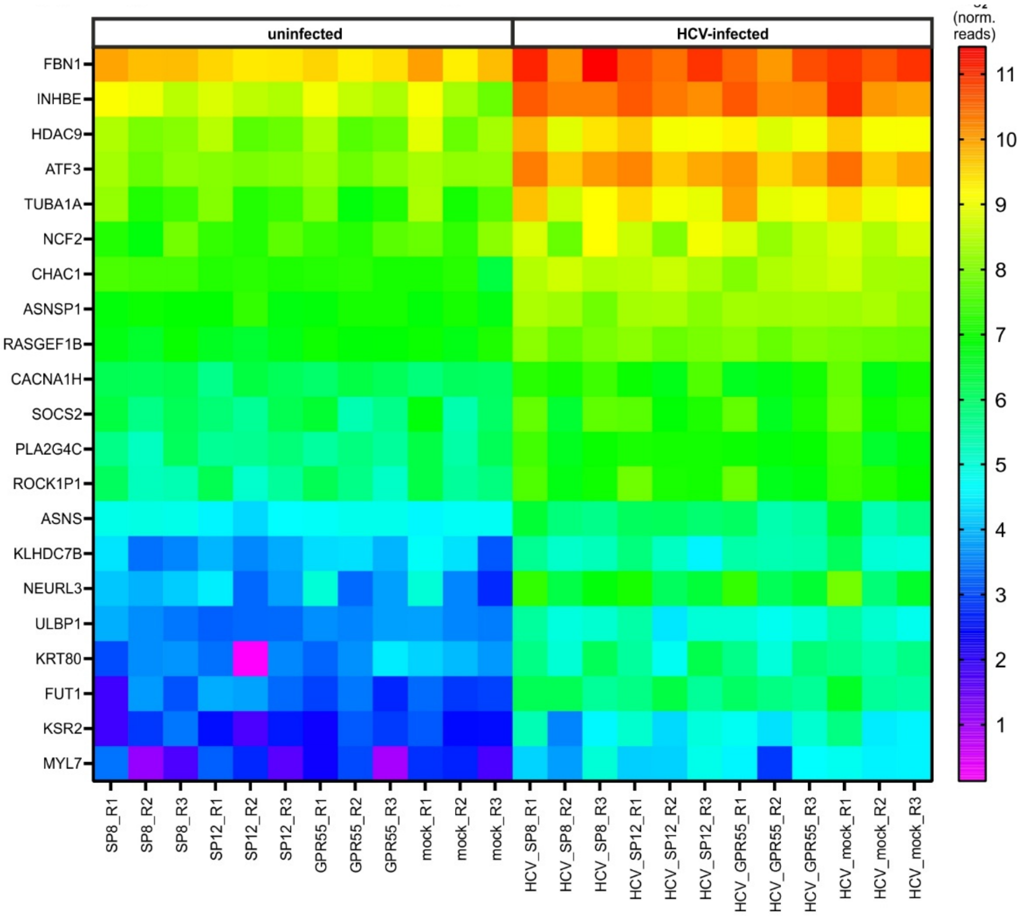

Supplementary Figure S7B-related to Figure 11B

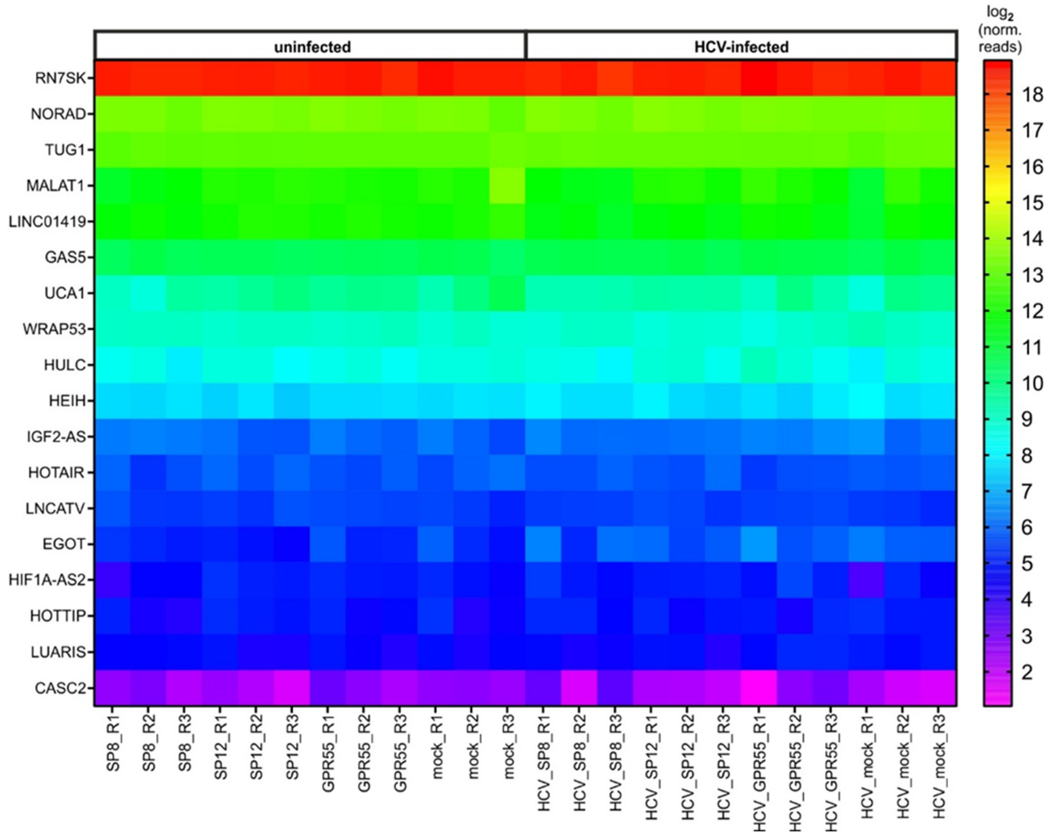

Supplementary Table S1: ***Lnc-ITM2C-1*** **splice variants information**

**Transcript**	**Transcript ID**	**Length**	**Genomic strand**	**Exon coordinates in Ref. gene Start-end**	**Chromosome coordinates**	**Database**
1	ENST00000447074.1GCSIR-203<br>lnc-ITM2C-1:1	557	+	Exon 1: 952-1421<br>Exon 2: 9834-9920	Chromosome 2: 230,886,500 - 230,895,468	Ensembl<br>LNCipedia
2	ENSG00000232520.1 | ENST00000415628.1<br>lnc-ITM2C-1:10	1,814	+	Exon 1: 949-1421<br>Exon 2: 9850-10057<br>Exon 3: 10411-11543	Chromosome 2: 230,886,497 – 230,897,091	LNCipedia<br>FANTOM CAT (stringent)
3	ENST00000455128.1GCSIR-204<br>lnc-ITM2C-1:2	544	+	Exon 1: 958-1421<br>Exon 2: 8447-8526	Chromosome 2: 230,886,506 - 230,894,074	Ensembl<br>LNCipedia
4	TCONS_00002732<br>lnc-ITM2C-1:3	1,892	+	Exon 1: 998-1421<br>Exon 2: 9850-10057<br>Exon 3: 10411-11542<br>Exon 4: 18841-18968	Chromosome 2: 230,886,546 – 230,904,516	LNCipedia
5	ENST00000415628.1GCSIR-201<br>lnc-ITM2C-1:4	1,765	+	Exon 1: 998-1421<br>Exon 2: 9850-10057<br>Exon 3: 10411-11543	Chromosome 2: 230,886,546 - 230,897,091	Ensembl<br>LNCipedia
6	ENST00000434807.1<br>TCONS_00003124<br>AC012507.3-002<br>OTTHUMT00000332599.1<br>NONHSAT077310<br>lnc-ITM2C-1:5	513	+	Exon 1: 1267-1421<br>Exon 2: 1833-2190	Chromosome 2: 230,886,815 – 230,887,738	LNCipedia
7	NONHSAT077306<br>lnc-ITM2C-1:6	1,020	+	Exon 1: 626-1421<br>Exon 2: 9834-10057	Chromosome 2: 230,886,174 – 230,895,605	LNCipedia
8	NR_038238.1<br>lnc-ITM2C-1:7	1,893	+	Exon 1: 998-1421<br>Exon 2: 9850-10057<br>Exon 3: 10411-11542<br>Exon 4: 18841-18969	Chromosome 2: 230,886,546 – 230,904,517	Ensembl<br>LNCipedia<br>NCBI
9	ENSG00000232520.1 | ENST00000455128.1<br>lnc-ITM2C-1:8	553	+	Exon 1: 949-1421<br>Exon 2: 8447-8526	Chromosome 2: 230886497 – 230,894,074	LNCipedia
10	ENSG00000232520.1 | ENST00000447074.1<br>lnc-ITM2C-1:9	560	+	Exon 1: 949-1421<br>Exon 2: 9834-9920	Chromosome 2: 230,886,497 – 230,895,468	LNCipedia
11	ENST00000658438.1-GCSIR-205	930	+	Exon 1: 926-1421<br>Exon 2: 1833-2266	Chromosome 2: 230,886,474 - 230,887,814	Ensembl
12	ENST00000663229.1-GCSIR-206	1,952	+	Exon 1: 961-1421<br>Exon 2: 10408-11542<br>Exon 3: 11622-11672<br>Exon 4: 18841-19145	Chromosome 2: 230,886,509 - 230,904,693	Ensembl
13	ENST00000670310.1-GCSIR-207	3,314	+	Exon 1: 933-1421<br>Exon 2: 9910-10057<br>Exon 3: 10408-13084	Chromosome 2: 230,886,481 - 230,898,632	Ensembl
14	ENST00000434807.2-GCSIR-202	658	+	Exon 1: 601-723<br>Exon 2: 1249-1421<br>Exon 3: 1833-2194	Chromosome 2: 230,886,149 - 230,887,742	Ensembl
15	ENST00000770323.1-GCSIR-208	382	+	Exon 1: 7953-8116<br>Exon 2: 8447-8664	Chromosome 2: 230,893,501 - 230,894,212	Ensembl

Supplementary Table S2: ***GPR55*** **splice variants information**

Transcript	Transcript ID	Length	Genomic strand	Exon coordinates in curated Ref. gene Start-end	Chromosome coordinates	Database
1	NM_005683.4; ENST00000650999.1 GPR55-206	3,844	-	Exon 1: 36142-36216<br>Exon 2: 50288-54056	Chromosome 2: 230,907,328 - 230,925,242	NCBI<br>Ensembl
2	XM_005246952.5	3,918	-	Exon 1: 36142-36290<br>Exon 2: 50288-54056	Chromosome 2: 230,907,328 - 230,925,242	NCBI
3	XM_011512175.4	4,196	-	Exon 1: 183-609<br>Exon 2: 50288-54056	Chromosome 2: 230,907,328 - 230,961,201	NCBI
4	XM_011512176.3	3,843	-	Exon 1: 33640-33713<br>Exon 2: 50288-54056	Chromosome 2: 230,907,328 - 230,927,744	NCBI
5	ENST00000392039.3 GPR55-201	4,069	-	Exon 1: 318-609<br>Exon 2: 50288-54064	Chromosome 2: 230,907,320 - 230,961,066	Ensembl
6	ENST00000392040.6 GPR55-202	3,852	-	Exon 1: 36142-36216<br>Exon 2: 50288-54064	Chromosome 2: 230,907,320 - 230,925,242	Ensembl
7	ENST00000438398.1 GPR55-203	919	-	Exon 1: 36158-36216<br>Exon 2: 50288-51147	Chromosome 2: 230,910,237 - 230,925,226	Ensembl
8	ENST00000444078.5 GPR55-204	2,527	-	Exon 1: 36158-36216<br>Exon 2: 50288-51762<br>Exon 3: 53074-54066	Chromosome 2: 230,907,318 - 230,925,226	Ensembl
9	ENST00000622008.5 GPR55-205	2,542	-	Exon 1: 36142-36216<br>Exon 2: 50288-51762<br>Exon 3: 53074-54065	Chromosome 2: 230,907,319 - 230,925,242	Ensembl
10	XM_054344567.1	4,125	-	Exon 1: 35936-36290<br>Exon 2: 50288-54056	Chromosome 2: 231,390,497 - 231,408,613	NCBI
11	XM_054344568.1	4,379	-	Exon 1: 1-609<br>Exon 2: 50288-54056	Chromosome 2: 231,390,497 - 231,444,798	NCBI
12	XM_054344569.1	3,846	-	Exon 1: 33638-33713<br>Exon 2: 50288-54056	Chromosome 2: 231,390,497 - 231,410,898	NCBI
13	ENST00000886146.2 GPR55-208	3,956	-	Exon 1: 36112-36290<br>Exon 2: 50288-54064	Chromosome 2: 230,907,320 - 230,925,272	Ensembl
14	ENST00000886145.1GPR55 -207	3,851	-	Exon 1: 33640-33713<br>Exon 2: 50288-54064	Chromosome 2: 230,907,320 - 230,927,744	Ensembl

Supplementary Table S3: Primer Sets for detection of *Lnc-ITM2C-1* splice variants

A) Successful primer sets used for downstream analysis (related to Fig.2)

PS name	Long primer name	Short primer name	Primer sequence 3′→5′	Amplicon size	Targeted splice variant	Design strategy	Detection Outcome	Selected for downstream use
PS3$_a$	Lnc-ITM2C-1 variant 7 unique- forward	Lv7u-F	GGATCTGGACTCTG CCATTGTCC	163 bp	7	unique exon region of splice variant 7	robust and specific	yes
	Lnc-ITM2C-1 variant 7 unique- Reverse	Lv7u-R	CCAGGTCACATGCA AAACACGTG					
PS9	Lnc-ITM2C-1 variants2/4/5/7/8-exon 2 common-Forward	Lv2/4/5/7/8ex2c-F	GGTTTTTTGACCTTG GCAATG	102 bp	2/4/5/7/8	constitutive exon 2-targeting	robust and specific	yes
	Lnc-ITM2C-1 variants2/4/5/7/8-exon 2common-Reverse	Lv2/4/5/7/8ex2c-R	GTGACCCTTGGTGG CTGTTTAT					
PS10$_f$	Lnc-ITM2C-1 variant 12 unique improved 3 Forward	Lv12ui3-F	GCTCAGGTAGACTG ACAACATAC	113 bp	12	unique exon region of splice variant 12	robust and specific	yes
	Lnc-ITM2C-1 variant 12 unique-improved 2 Reverse	Lv12ui2-R	GTGGGATGAAATTG ATGGTTAAGAG					

B) Successful primer sets used for independent confirmatory validation

PS name	Long primer name	Short primer name	Primer sequence 3′→5′	Amplicon size	Targeted splice variant	Design strategy	Detection Outcome	Selected for downstream use
PS5	Lnc-ITM2C-1 variants2/4/5/8/12/13-exon 3 common-Forward	Lv2/4/5/8/12/13 ex3c-F	GATTCTGTCTCATCC AATCAAGACT	123 bp	2/4/5/8/12/1 3	constitutive exon 3-targeting	robust and specific	yes
	Lnc-ITM2C-1 variants2/4/5/8/12/13exon 3 common-Reverse	Lv2/4/5/8/12/13 ex3c-R	GTTGTGCTGAGGAT TCTGGGT					
PS10$_a$	Lnc-ITM2C-1 variant 12 unique improved 1-Forward	Lv12ui1-F	GGCAGCTCAGGTAG ACTGAC	101 bp	12	unique exon region of splice variant 12	robust and specific	yes
	Lnc-ITM2C-1- variant 12 unique improved 1-Reverse	Lv12ui1-R	GGTTAAGAGATCTG TCAGCTATTG					
PS10$_e$	Lnc-ITM2C-1 variant 12 unique improved 3-Forward	Lv12ui3-F	GCTCAGGTAGACTG ACAACATAC	97 bp	12	unique exon region of splice variant 12	robust and specific	yes
	Lnc-ITM2C-1 variant 12 unique improved 1-Reverse	Lv12ui1-R	GGTTAAGAGATCTG TCAGCTATTG					

C) Suboptimal primer sets producing the expected product with additional nonspecific amplification

PS name	Long primer name	Short primer name	Primer sequence 3′→5′	Amplicon size	Targeted splice variant	Design strategy	Detection Outcome	Selected for downstream use
PS2$_b$	Lnc-ITM2C-1 all variants except 6/14/15 exon 1 common- Forward	Lexc.v6/14/15ex1c-F	TGGAAGAGAGGCTT TGAGTCTAGGG	67 bp	All except 6/14/15	constitutive exon 1-targeting	partially robust and specific	no
	Lnc-ITM2C-1 all variants except 6/14/15 exon 1 common improved 1-Reverse	Lexc.v6/14/15ex1ci 1-R	CCCTTCTAGGACTG CACCCC					
PS2$_c$	Lnc-ITM2C-1 all variants except 6/14/15 exon 1 common- Forward	Lexc.v6/14/15ex1c-F	TGGAAGAGAGGCTT TGAGTCTAGGG	115 bp	All except 6/14/15	constitutive exon 1-targeting	partially robust and specific	no
	Lnc-ITM2C-1 all variants except 6/14/15 exon 1 common improved 2 -Reverse	Lexc.v6/14/15-R	ATGCAGCAAGGATA CGGAGATGC					
PS2$_d$	Lnc-ITM2C-1 all variants except 6/14/15 exon 1 common improved 1-Forward	Lexc.v6/14/15ex1ci 1-F	CGTATCCTTGCTGCA TGCTG	122 bp	All except 6/14/15	constitutive exon 1-targeting	partially robust and specific	no
	Lnc-ITM2C-1 all variants except 15 exon 1 common-Reverse	Lexc.v15ex1c-R	CTTCCAGTGACAGG GCCCTTTG					

PS4$_b$	Lnc-ITM2C-1 variant 13 unique improved 1 – forward	Lv13ui1-F	GCTGAAGGTAGCAG CTCCAGG	199 bp	13	unique exon 3 of splice variant 13	partially robust and specific	no
	Lnc-ITM2C-1 variant 13 unique improved 2-Reverse	Lv13ui2-R	GGTCTCTGTCTCCTA CCACCGTTGTC					
PS4$_c$	Lnc-ITM2C-1 variant 13 unique improved 2 – Forward	Lv13ui2-F	CTGAAGGTAGCAGC TCCAGG	198 bp	13	unique exon 3 of splice variant 13	partially robust and specific	no
	Lnc-ITM2C-1 variant 13 unique improved 2-Reverse	Lv13ui2-R	GGTCTCTGTCTCCTA CCACCGTTGTC					
PS4$_d$	Lnc-ITM2C-1 variant 13 unique improved 3 – Forward	Lv13ui3-F	GAGTGTTGTCTAGG AGAAGCCCC	180 bp	13	unique exon 3 of splice variant 13	partially robust and specific	no
	Lnc-ITM2C-1 variant 13 unique improved 3-Reverse	Lv13ui3-R	GTGGTGGTGTGCTT GTCATCCC					
PS10	Lnc-ITM2C-1 variant 12 unique- Forward	Lv12u-F	GGTAGACTGACAAC ATACTTGCC	133 bp	12	unique exon region of splice variant 12	partially robust and specific	no
	Lnc-ITM2C-1 variant 12 unique- Reverse	Lv12u-R	GGACTATTTCAATTA TATGAACAAAGTGG G					
PS10$_b$	Lnc-ITM2C-1 variant 12 unique improved 1-Forward	Lv12ui1-F	GGCAGCTCAGGTAG ACTGAC	117 bp	12	unique exon region of splice variant 12	partially robust and specific	no
	Lnc-ITM2C-1 variant 12 unique improved 2-Reverse	Lv12ui2-R	GTGGGATGAAATTG ATGGTTAAGAG					
PS10$_c$	Lnc-ITM2C-1 variant 12 unique-improved 2-Forward	Lv12ui2-F	CAGTGTTTTATTATG GCAGCTCAGGTAG	115 bp	12	unique exon region of splice variant 12	partially robust and specific	no
	Lnc-ITM2C-1 variant 12 unique-improved 1-Reverse	Lv12ui1-R	GGTTAAGAGATCTG TCAGCTATTG					
PS10$_d$	Lnc-ITM2C-1 variant 12 unique-improved 2-Forward	Lv12ui2-F	CAGTGTTTTATTATG GCAGCTCAGGTAG	131 bp	12	unique exon region of splice variant 12	partially robust and specific	no
	Lnc-ITM2C-1 variant 12 unique-improved 2-Reverse	Lv12ui2-R	GTGGGATGAAATTG ATGGTTAAGAG					

D) Unsuccessful primer sets

PS name	Long primer name	Short primer name	Primer sequence 3′→5′	Amplicon size	Targeted splice variant	Design strategy	Detection Outcome	Selected for downstream use
PS1	Lnc-ITM2C-1 all variants except 4/5/6/8/14/15exon 1 common-Forward	Lexcp.v4/5/6/8/14/ 15ex1c-F	GGAACCATTTGAGC TTTGAAGGTGGG	365 bp	All except 4/5/6/8/14/1 5	constitutive exon 1-targeting	not specific	no
	lnc-ITM2C-1 all variants except 15 exon 1common-Reverse	Lexc.v15ex1c-R	CTTCCAGTGACAGG GCCCTTTG					
PS1$_a$	Lnc-ITM2C-1 all variants except 4/5/6/8/14/15 exon 1 common-Forward	Lexcp.v4/5/6/8/14/ 15ex1c-F	GGAACCATTTGAGC TTTGAAGGTGGG	211 bp	All except 4/5/6/8/14/1 5	constitutive exon 1-targeting	not specific	no
	Lnc-ITM2C-1 all variants except 6/14/15 exon 1 common improved1-Reverse	Lexc.v6/14/15ex1ci 1-R	CCCTTCTAGGACTG CACCCC					
PS1$_b$	Lnc-ITM2C-1 all variants except 4/5/6/8/14/15 exon 1 common-Forward	Lexcp.v4/5/6/8/14/ 15ex1c-F	GGAACCATTTGAGC TTTGAAGGTGGG	259 bp	All except 4/5/6/8/14/1 5	constitutive exon 1-targeting	not specific	no
	Lnc-ITM2C-1 all variants except 6/14/15 exon 1 common improved2 - Reverse	Lexc.v6/14/15ex1ci 2-R	ATGCAGCAAGGATA CGGAGATGC					
PS1$_c$	Lnc-ITM2C-1 all variants except 4/5/6/8/14/15 exon 1 common-Forward	Lexcp.v4/5/6/8/14/ 15ex1c-F	GGAACCATTTGAGC TTTGAAGGTGGG	423 bp	All except 4/5/6/8/14/1 5	constitutive exon 1-targeting	not specific	no
	Lnc-ITM2C-1 all variants except 15 exon 1 common improved1-Reverse	Lexc.v15ex1ci1-R	CCATCAGTCCTCCG TTAGGCG					
PS2	Lnc-ITM2C-1 primer detecting all variants	Lexc.v6/14/15ex1c-F	TGGAAGAGAGGCTT TGAGTCTAGGG	221 bp	All except 6/14/15	constitutive exon 1-targeting	not specific	no

	except 6/14/15 exon 1 common- Forward							
	Lnc-ITM2C-1 all variants except 15 exon 1common-Reverse	Lexc.v15ex1c-R	CTTCCAGTGACAGG GCCCTTTG					
PS2$_{a}$	Lnc-ITM2C-1 primer detecting all variants except 6/14/15 exon 1 common- Forward	Lexc.v6/14/15ex1c-F	TGGAAGAGAGGCTT TGAGTCTAGGG	279 bp	All except 6/14/15	constitutive exon 1-targeting	not specific	no
	Lnc-ITM2C-1 all variants except 15 exon1 common improved1-Reverse	Lexc.v15ex1ci1-R	CCATCAGTCCTCCG TTAGGCG					
PS2$_{e}$	lnc-ITM2C-1 all variants except 6/14/15 exon 1 common improved 1-Forward	Lexc.v6/14/15ex1ci 1-F	CGTATCCTTGCTGCA TGCTG	180 bp	All except 6/14/15	constitutive exon 1-targeting	not specific	no
	Lnc-ITM2C-1 all variants except 15 exon1 common improved1-Reverse	Lexc.v15ex1ci1-R	CCATCAGTCCTCCG TTAGGCG					
PS3	Lnc-ITM2C-1 variant 7 unique- Forward	Lv7u-F	GGATCTGGACTCTG CCATTGTCC	782 bp	7	unique exon region of splice variant 7	not specific	no
	Lnc-ITM2C-1 variants2/4/5/7/8-exon2 common-Reverse	Lv2/4/5/7/8ex2c-R	GTGACCCTTGGTGG CTGTTTAT					
PS4	Lnc-ITM2C-1 variant 13 unique- Forward	Lv13u-F	GGACATAGACTGCA CAGATTCAGCC	553 bp	13	unique exon 3 of splice variant 13	not specific	no
	Lnc-ITM2C-1 – primer targets variant 13 unique- Reverse	Lv13u-R	CACTATCTCTGCTGG CCTCTCCA					
PS4$_{a}$	Lnc-ITM2C-1 variant 13 unique- Forward	Lv13u-F	GGACATAGACTGCA CAGATTCAGCC	622 bp	13	unique exon 3 of splice variant 13	not specific	no
	Lnc-ITM2C-1 – primer targets variant 13 unique improved 1- Reverse	Lv13ui1-R	TATTGGCTCTGCTCT ATCCTTCCTGC					
PS6	Lnc-ITM2C-1 variant 2/4/5/8 exon1-2 common -Forward	Lv2/4/5/8ex1-2c-F	GCAGCAGTGAGGTT TTTTGACCTTGG	228 bp	2/4/5/8	constitutive exon 2-targeting/exon-exon junction-spanning	not specific	no
	Lnc-ITM2C-1 variant 2/4/5/8 exon 1-2 common -Reverse	Lv2/4/5/8ex1-2c-R	CCTTAGCCCCGTGT CCTTGTC					
PS6$_{a}$	Lnc-ITM2C-1 variant 2/4/5/8 exon 1-2 common improved 1-Forward	Lv2/4/5/8ex1-2ci1-F	ATAAACAGCCACCA AGGGTCAC	145 bp	2/4/5/8	constitutive exon 2-targeting/exon-exon junction-spanning	not specific	no
	Lnc-ITM2C-1 variant 2/4/5/8 exon1-2 common improved 1-Reverse	Lv2/4/5/8ex1-2ci1-R	GGACAGTCCTTAGC CCCGTG					
PS6$_{b}$	Lnc-ITM2C-1 variant 2/4/5/8 exon1-2 common -Forward	Lv2/4/5/8ex1-2c-F	GCAGCAGTGAGGTT TTTTGACCTTGG	235 bp	2/4/5/8	constitutive exon 2-targeting/exon-exon junction-spanning	not specific	no
	Lnc-ITM2C-1 variant 2/4/5/8 exon1-2 common improved 1-Reverse	Lv2/4/5/8ex1-2ci1-R	GGACAGTCCTTAGC CCCGTG					
PS7	Lnc-ITM2C-1 variant 6/11/14 exon 2-Forward	Lv6/11/14ex2-F	CAGCAGTGAGGGG CCAATCATC	232 bp	6/11/14	exon-exon junction-spanning	not specific	no
	Lnc-ITM2C-1 variant 6/11/14 exon2 -Reverse	Lv6/11/14ex2-R	GTTGCACTCTGACC TTACAATGGC					
PS8	Lnc-ITM2C-1 all variants except 4/5/6/8/14/15exon 1 common-Forward	Lexcp.v4/5/6/8/14/15ex1c-F	GGAACCATTTGAGC TTTGAAGGTGGG	670 bp	11	exon-spanning/ constitutive exon 2-targeting	not specific	no
	Lnc-ITM2C-1 variants 6/11/14exon 2-Reverse	Lv6/11/14ex2-R	GTTGCACTCTGACC TTACAATGGC					
PS8$_{a}$	Lnc-ITM2C-1 all variants except 6/14/15 exon 1 common improved 1-Forward	Lexc.v6/14/15ex1ci 1-F	CGTATCCTTGCTGCA TGCTG	287 bp	11	exon-spanning/ constitutive exon 2-targeting	not specific	no
	Lnc-ITM2C-1 variants 6/11/14 exon 2 improved 1-Reverse	Lv6/11/14ex2i1-R	AGTAGGTGGCTGGT GTCTCC					

- Lowercase letters indicate optimization variants of the same primer set($_{a,b,c,d,e,f}$).
- c=common
- i=improved
- ex=exon
- excp. =except
- v=variant
- L=Lnc-ITM2C-1

Supplementary Table S4: Primer Sets for detection of *GPR55* splice variants

A) Successful primer sets used for downstream analysis (related to Fig.2)

PS name	Long primer name	Short primer name	Primer sequence 3′→5′	Amplicon size	Targeted splice variant	Design strategy	Detection outcome	Selected for downstream use
PS5	GPR55 all variants-Forward	Gva-F	AAGAGAGTGAAAG GAGCCCCGTCT	136 bp	all	constitutive exon 2 targeting	robust and specific	yes
	GPR55 all variants-Reverse	Gva-R	GGGATGTGGACTGC AAACTGTAGG					

B) Successful primer sets used for independent confirmatory validation

PS name	Long primer name	Short primer name	Primer sequence 3′→5′	Amplicon size	Targeted splice variant	Design strategy	Detection outcome	Selected for downstream use
PS4	GPR55 variant 3/11-Forward	Gv3/11-F	GCCTTTCTCTTTTT CTCCCCCGA	111 bp	3/11	unique exon region of exon 1 of splice variant 3	robust and specific- confirmed results of PS3	Initially, yes
	GPR55 variant 3/11-Reverse	Gv3/11-R	CAAAGGACACTG CCTTACCAGG					
PS6	GPR55 all variants validation1-Forward	Gvavali1-F	GAAAACCCTACA GTTTGCAGTCC	123 bp	all	constitutive exon 2 targeting	robust and specific	Initially yes
	GPR55 all variants validation1-Reverse	Gvavali1-R	GAGGTGGCAGCA TAATCGGG					
PS7	GPR55 all variants validation2-Forward	Gvavali2-F	GGTGGAGTGCCTT TACTTCGT	97 bp	all	constitutive exon 2 targeting	robust and specific	Initially yes
	GPR55 all variants validation2 except 10/11/12-Reverse	Gvaexcp.10/11/12vali2-R	CAGTAGCGGGTA ACGGATGG					
PS8	GPR55 all variants validation3-Forward	Gvavali3-F	GGACCGGAAGCA TCCCTATCT	87 bp	all	constitutive exon 2 targeting	robust and specific	Initially yes
	GPR55 all variants validation3 except 10/11/12-Reverse	Gvaexcp.10/11/12vali3-R	CGCTCCAGGTATC ATCAGACA					
PS12	GPR55 all variants except 5/7/8/9-Forward	Gvaexcp.5/7/8/9-F	CAGCTGGCCTAA GAGCCATGGAC	243 bp	1/2/3/4/6	targeting the unique intron sequence of splice variant 8,9	robust and specific	no
	GPR55 all variants except 5/7 detect 8/9-Reverse	Gvaexcp. 5/7dev8/9-R	TCTGTGGCTGTGG TTCTTAGAGG					

C) Suboptimal primer sets producing the expected product with additional nonspecific amplification

PS name	Long primer name	Short primer name	Primer sequence 3′→5′	Amplicon size	Targeted splice variant	Design strategy	Detection outcome	Selected for downstream use
PS3	GPR55 variants 3/11-Forward	Gv3/11-F	GCCTTTCTCTTTTTC TCCCCCGA	365 bp	3/11	unique exon region of exon 1 of splice variant 3/11	partially robust and specific	no
	GPR55 variants 3/5/11-Reverse	Gv3/5/11-R	GCTGGAGGCTGAGT GTTTGGGC					
PS10	GPR55 variants 1/6/7/8/9 exon-exon-Forward	Gv1/6/7/8/9ex-ex-F	CCACCAACAGTTGT GATTCAATGGGC	230 bp	1/6/7/8/9	exon-exon junction	partially robust and specific	no
	GPR55 all variants-Reverse	Gva-R	GGGATGTGGACTGC AAACTGTAGG					

D) Unsuccessful primer sets

PS name	Long primer name	Short primer name	Primer sequence 3′→5′	Amplicon size	Targeted splice variant	Design strategy	Detection outcome	Selected for downstream use
PS1	GPR55 variants 1/2/6/8/7/9/10-Forward	Gv1/2/6/8/7/9/10-F	GAAATGACATCTC TCAGCCCTC	266 bp (for splice 1/6/7/8/9) 340 bp (for splice 2)	1/2/6/7/8/9	intron spanning (exon1-2)	not specific	no
	GPR55 all variants-Reverse	Gva-R	GGGATGTGGACT GCAAACTGTAGG					
PS2	GPR55 variants 2/10-Forward	Gv2/10-F	AACAGCCTCTGG GCCATTTAGAGA	292 bp	2	intron spanning (exon 1-2)/ unique region in exon 1 of splice variant 2	not specific	no
	GPR55 all variants-Reverse	Gva-R	GGGATGTGGACT GCAAACTGTAGG					

PS9	GPR55 all variants detect 8/9 except 5/7-Forward	Gvade8/9excp.5/7-F	CATTTTCCTGTTCGACTGGGAGGTTC	199 bp	8/9	intron spanning (exon 2-3)	not specific	no
	GPR55 all variants except 5/7 detect 8/9-Reverse	Gvaexcp. 5/7dev8/9-R	TCTGTGGCTGTGGTTCTTAGAGG					
PS11	GPR55 variants 8/9 exon-exon -Forward	Gva8/9ex-ex-F	GAGAGAGAGAGAAAAGAGGTGGATGG	163 bp	8/9	exon-exon junction	not specific	no
	GPR55 all variants except 5/7 detect 8/9-Reverse	Gvaexcp. 5/7dev8/9-R	TCTGTGGCTGTGGTTCTTAGAGG					
PS13	GPR55 variants 4/12-Forward	Gv4/12-F	CTTGTTCCTTGCCCTAGAAGAAGC	287 bp	4/12	unique exon region of splice variant 4,12	not specific	no
	GPR55 all variants-Reverse	Gva-R	GGGATGTGGACTGCAAACTGTAGG					
PS14	GPR55 variants 4/12 improved 1-Forward	Gv4/12im1-F	GCTGCTCCACTGGTGAAGTCGAAG	244 bp	4/12	unique exon region of splice variant 4,12	not specific	no
	GPR55 all variants-Reverse	Gva-R	GGGATGTGGACTGCAAACTGTAGG					

- Lowercase letters indicate optimization variants of the same primer set(a,b,c,d,e,f).
- PS = primer set
- F = forward
- R = reverse
- c = common
- a = all
- im = improved
- ex = exon
- excp. = except
- v = variant
- G = GPR55
- de = detect
- vali = validation

Supplementary Table S5A-related to Discossion section

Current study	exonic	non_exonic
	71,2 %	28,8 %
Previous study	exonic	non_exonic
	81,7 %	18,3 %

Supplementary Table S5B-related to Discossion section and Figure S11C

	present study			previous study		
	> 0 reads	# samples	%	> 0 reads	# samples	%
**RN7SK**	24	24	100,0	22	22	100,0
**NORAD/LINC00657**	24	24	100,0	22	22	100,0
**TUG1**	24	24	100,0	22	22	100,0
**MALAT1**	24	24	100,0	22	22	100,0
**LINC01419**	24	24	100,0	22	22	100,0
**GAS5**	24	24	100,0	22	22	100,0
**UCA1**	24	24	100,0	22	22	100,0
**WRAP53**	24	24	100,0	22	22	100,0
**HULC**	24	24	100,0	22	22	100,0
**HEIH**	24	24	100,0	22	22	100,0
**IGF2-AS**	24	24	100,0	22	22	100,0
**HOTAIR**	24	24	100,0	22	22	100,0
**LNCATV**	24	24	100,0	0	22	0,0
**EGOT**	24	24	100,0	19	22	86,4
**HIF1A-AS1**	24	24	100,0	21	22	95,5
**HOTTIP**	24	24	100,0	0	22	0,0
**LUARIS**	24	24	100,0	0	22	0,0
**CASC2**	24	24	100,0	18	22	81,8
**GRP55**	24	24	100,0	16	22	72,7
**GCSIR/Lnc-ITM2C-1**	11	12	91,7	0	22	0,0

(A) Estimation of exonic vs. non-exonic reads in annotated genes from the RNA-Seq experiments in this study (upper part, blue) and from our previous study (lower part, red; that study is also referred to in Fig. 3E).

(B) The number of biological replicates from the RNA-Seq experiments in this study (blue) and from our previous study (red; that study is also referred to in Fig. 3E) for which a read count of > 0 was obtained for the respective lncRNAs, the corresponding number of total replicates (# samples) and the percentage (%) of samples with > 0 reads. For GCSIR/Lnc-ITM2C-1, only those replicates were counted in which Lnc-ITM2C-1 was not overexpressed.
