# Supplementary material for: Transcriptome Analysis of Hepatitis C Virus-Infected Hepatoma Cells Following Stringent Removal of Genomic DNA
Source: Viruses. 2026 Oct 20;18(10):1167. doi: 10.3390/v18101167 (PMC13709437; doi:10.3390/v18101167)
Supplement: Supplementary file 1 [file viruses-18-01167-s001.zip › Khajouei_Transcripts and Chromosome Nt numbers - Lnc-ITM2C-1 and GPR55.pdf]

1) *Lnc-ITM2C-1* splice variants information

Transcript	Transcript ID	Length	Genomic strand	Exon coordinates in Ref. gene Start-end	Chromosome coordinates	Database
1	ENST00000447074.1GCSIR-203<br>lnc-ITM2C-1:1	557	+	Exon 1: 952-1421<br>Exon 2: 9834-9920	Chromosome 2: 230,886,500 - 230,895,468	Ensembl<br>LNCipedia
2	ENSG00000232520.1|ENST00000415628.1<br>lnc-ITM2C-1:10	1,814	+	Exon 1: 949-1421<br>Exon 2: 9850-10057<br>Exon 3: 10411-11543	Chromosome 2: 230,886,497 – 230,897,091	LNCipedia<br>FANTOM CAT (stringent)
3	ENST00000455128.1GCSIR-204<br>lnc-ITM2C-1:2	544	+	Exon 1: 958-1421<br>Exon 2: 8447-8526	Chromosome 2: 230,886,506 - 230,894,074	Ensembl<br>LNCipedia
4	TCONS_00002732<br>lnc-ITM2C-1:3	1,892	+	Exon 1: 998-1421<br>Exon 2: 9850-10057<br>Exon 3: 10411-11542<br>Exon 4: 18841-18968	Chromosome 2: 230,886,546 – 230,904,516	LNCipedia
5	ENST00000415628.1GCSIR-201<br>lnc-ITM2C-1:4	1,765	+	Exon 1: 998-1421<br>Exon 2: 9850-10057<br>Exon 3: 10411-11543	Chromosome 2: 230,886,546 - 230,897,091	Ensembl<br>LNCipedia
6	ENST00000434807.1<br>TCONS_00003124<br>AC012507.3-002<br>OTTHUMT00000332599.1<br>NONHSAT077310<br>lnc-ITM2C-1:5	513	+	Exon 1: 1267-1421<br>Exon 2: 1833-2190	Chromosome 2: 230,886,815 – 230,887,738	LNCipedia
7	NONHSAT077306<br>lnc-ITM2C-1:6	1,020	+	Exon 1: 626-1421<br>Exon 2: 9834-10057	Chromosome 2: 230,886,174 – 230,895,605	LNCipedia
8	NR_038238.1<br>lnc-ITM2C-1:7	1,893	+	Exon 1: 998-1421<br>Exon 2: 9850-10057<br>Exon 3: 10411-11542<br>Exon 4: 18841-18969	Chromosome 2: 230,886,546 – 230,904,517	Ensembl<br>LNCipedia<br>NCBI
9	ENSG00000232520.1|ENST00000455128.1<br>lnc-ITM2C-1:8	553	+	Exon 1: 949-1421<br>Exon 2: 8447-8526	Chromosome 2: 230886497 – 230,894,074	LNCipedia
10	ENSG00000232520.1|ENST00000447074.1<br>lnc-ITM2C-1:9	560	+	Exon 1: 949-1421<br>Exon 2: 9834-9920	Chromosome 2: 230,886,497 – 230,895,468	LNCipedia
11	ENST00000658438.1-GCSIR-205	930	+	Exon 1: 926-1421<br>Exon 2: 1833-2266	Chromosome 2: 230,886,474 - 230,887,814	Ensembl
12	ENST00000663229.1-GCSIR-206	1,952	+	Exon 1: 961-1421<br>Exon 2: 10408-11542<br>Exon 3: 11622-11672<br>Exon 4: 18841-19145	Chromosome 2: 230,886,509 - 230,904,693	Ensembl
13	ENST00000670310.1-GCSIR-207	3,314	+	Exon 1: 933-1421<br>Exon 2: 9910-10057<br>Exon 3: 10408-13084	Chromosome 2: 230,886,481 - 230,898,632	Ensembl
14	ENST00000434807.2-GCSIR-202	658	+	Exon 1: 601-723<br>Exon 2: 1249-1421<br>Exon 3: 1833-2194	Chromosome 2: 230,886,149 - 230,887,742	Ensembl
15	ENST00000770323.1-GCSIR-208	382	+	Exon 1: 7953-8116<br>Exon 2: 8447-8664	Chromosome 2: 230,893,501 - 230,894,212	Ensembl

2) ***GPR55*** **splice variants information**

Transcript	Transcript ID	Length	Genomic strand	Exon coordinates in curated Ref. gene Start-end	Chromosome coordinates	Database
1	NM_005683.4;<br>ENST00000650999.1<br>GPR55-206	3,844	-	Exon 1: 36142-36216<br>Exon 2: 50288-54056	Chromosome 2:<br>230,907,328 -<br>230,925,242	NCBI<br>Ensembl
2	XM_005246952.5	3,918	-	Exon 1: 36142-36290<br>Exon 2: 50288-54056	Chromosome 2:<br>230,907,328 -<br>230,925,242	NCBI
3	XM_011512175.4	4,196	-	Exon 1: 183-609<br>Exon 2: 50288-54056	Chromosome 2:<br>230,907,328 -<br>230,961,201	NCBI
4	XM_011512176.3	3,843	-	Exon 1: 33640-33713<br>Exon 2: 50288-54056	Chromosome 2:<br>230,907,328 -<br>230,927,744	NCBI
5	ENST00000392039.3<br>GPR55-201	4,069	-	Exon 1: 318-609<br>Exon 2: 50288-54064	Chromosome 2:<br>230,907,320 -<br>230,961,066	Ensembl
6	ENST00000392040.6<br>GPR55-202	3,852	-	Exon 1: 36142-36216<br>Exon 2: 50288-54064	Chromosome 2:<br>230,907,320 -<br>230,925,242	Ensembl
7	ENST00000438398.1<br>GPR55-203	919	-	Exon 1: 36158-36216<br>Exon 2: 50288-51147	Chromosome 2:<br>230,910,237 -<br>230,925,226	Ensembl
8	ENST00000444078.5<br>GPR55-204	2,527	-	Exon 1: 36158-36216<br>Exon 2: 50288-51762<br>Exon 3: 53074-54066	Chromosome 2:<br>230,907,318 -<br>230,925,226	Ensembl
9	ENST00000622008.5<br>GPR55-205	2,542	-	Exon 1: 36142-36216<br>Exon 2: 50288-51762<br>Exon 3: 53074-54065	Chromosome 2:<br>230,907,319 -<br>230,925,242	Ensembl
10	XM_054344567.1	4,125	-	Exon 1: 35936-36290<br>Exon 2: 50288-54056	Chromosome 2:<br>231,390,497 -<br>231,408,613	NCBI
11	XM_054344568.1	4,379	-	Exon 1: 1-609<br>Exon 2: 50288-54056	Chromosome 2:<br>231,390,497 -<br>231,444,798	NCBI
12	XM_054344569.1	3,846	-	Exon 1: 33638-33713<br>Exon 2: 50288-54056	Chromosome 2:<br>231,390,497 -<br>231,410,898	NCBI
13	ENST00000886146.2<br>GPR55-208	3,956	-	Exon 1: 36112-36290<br>Exon 2: 50288-54064	Chromosome 2:<br>230,907,320 -<br>230,925,272	Ensembl
14	ENST00000886145.1GPR<br>55-207	3,851	-	Exon 1: 33640-33713<br>Exon 2: 50288-54064	Chromosome 2:<br>230,907,320 -<br>230,927,744	Ensembl
